# Natural product preferentially targets redox and metabolic adaptations and aberrantly active STAT3 to inhibit breast tumor growth in vivo

**DOI:** 10.1038/s41419-022-05477-2

**Published:** 2022-12-06

**Authors:** Yinsong Zhu, Peibin Yue, Cody F. Dickinson, Justin K. Yang, Kyrstin Datanagan, Ning Zhai, Yi Zhang, Gabriella Miklossy, Francisco Lopez-Tapia, Marcus A. Tius, James Turkson

**Affiliations:** 1 Division of Medical Oncology, Department of Medicine, Cedars-Sinai Medical Center, 8700 Beverly Boulevard, Los Angeles, CA 90048 USA; 2 Cedars-Sinai Cancer, Cedars-Sinai Medical Center, 8700 Beverly Boulevard, Los Angeles, CA 90048 USA; 3 Department of Chemistry, University of Hawaii, Manoa, 2545 McCarthy Mall, Honolulu, HI 96825 USA; 4 Biobank and Research Pathology Resource, Academic Affairs and Research Administration, Cedars-Sinai Medical Center, 8700 Beverly Boulevard, Los Angeles, CA 90048 USA; 5 Cancer Biology Program, University of Hawaii Cancer Center, 701 Ilalo Street, Honolulu, HI 96813 USA

**Keywords:** Natural products, Breast cancer

## Abstract

Dysregulated gene expression programs and redox and metabolic adaptations allow cancer cells to survive under high oxidative burden. These mechanisms also represent therapeutic vulnerabilities. Using triple-negative breast cancer (TNBC) as a model, we show that compared to normal human breast epithelial cells, the TNBC cells, MDA-MB-231 and MDA-MB-468 that harbor constitutively active STAT3 also express higher glucose-6-phosphate dehydrogenase (G6PD), thioredoxin reductase (TrxR)1, NADPH, and GSH levels for survival. Present studies discover that the natural product, R001, targets these adaptation mechanisms. Treatment of TNBC cells with R001 inhibited constitutively active STAT3, STAT3-regulated gene expression, and the functions of G6PD and TrxR1. Consequently, in the TNBC, but not normal cells, R001 suppressed GSH levels, but raised NADPH levels, reflective of a loss of mitochondrial respiration and which led to reactive oxygen species (ROS) induction, all of which led to loss of viable cells and inhibition of anchorage-dependent and independent growth. R001 treatment further led to early pyroptosis and late DNA damage, cell cycle arrest, and apoptosis only in the TNBC cells. Oral administration of 5 mg/kg R001 inhibited MDA-MB-468 xenografts growth in mice, with reduced pY705-STAT3, G6PD, TrxR1, and GSH levels. R001 serves as a therapeutic entity that targets the vulnerabilities of TNBC cells to inhibit tumor growth in vivo.

## Introduction

Genetic, epigenetic, and other intracellular aberrations in cancer cells promote an oxidative stress environment that is a burden to tumor cells [1]. To survive the deleterious effects of the oxidative stress, tumor cells have adapted to utilizing the cellular biochemical and molecular anti-oxidant machinery, and thus have high dependency on cellular anti-oxidant molecules [1]. Glutathione (GSH) is an anti-oxidant that is essential to the survival of cells in serving as a buffer against oxidative stress [1, 2]. The biochemical pathway that regenerates GSH (reduced) from the oxidized glutathione (GSSG) utilizes the reducing equivalent from nicotinamide adenine dinucleotide phosphate (NADPH) [1, 2]. The generation of NADPH is linked to other biochemical pathways, including a step in glycolysis and the pentose-phosphate pathway, which are dependent on glucose-6-phosphate dehydrogenase (G6PD) [1], which has been implicated in cancer progression. Its high expression predicts high risk of tumor recurrence and worse progression-free survival and overall survival [3, 4]. Another important biochemical pathway for maintaining redox homeostasis is the thioredoxin (TRX)-thioredoxin reductase (TrxR) system [1]. The high expression of both thioredoxin 1 (Trx1) and thioredoxin reductase 1 (TrxR1) is observed in several human cancers, including breast cancer, and the over-expression of Trx1 in MDA-MB-231 breast cancer cell lines promoted an aggressive phenotype [5].

Among the signaling pathways that are implicated in human cancers is the signal transducer and activator of transcription (STAT)3. Aberrantly active STAT3 promotes cancer progression in part by dysregulation of gene expression to support uncontrolled cell growth and survival, angiogenesis, and metastasis and by suppressing tumor immune surveillance [6–9]. The inhibition of constitutively active STAT3 suppressed tumor cell growth in vitro and tumor growth in vivo [8, 10–22].

Natural products have been an inspiration for developing new treatment modalities. They have the potential to modulate multiple targets at low potency, hence their reduced toxicity and their overall beneficial therapeutic responses. Frequently, there is minimal understanding of the underlying mechanisms for their biological effects. We previously reported on the hirsutinolide natural products, including **6**, **7**, **10**, **20**, and **22**, from the *Vernonia cinerea* plant, which inhibited human glioblastoma growth in vivo [23].

We herein report that human TNBC, prostate, pancreatic, and non-small cell lung cancer cells are more sensitive than normal cells to the hirsutinolide natural product R001 (compound **6**), which preferentially decreased the viable cell numbers, colony formation, growth in 3D-matrigel, or migration of the tumor cells. Comparing TNBC as a model to normal human breast epithelial MCF-10A cells, we observed that the TNBC cells, MDA-MB-231 and MDA-MB-468 have higher reduced nicotinamide adenine dinucleotide phosphate (NADPH) and glutathione (GSH) levels, and higher glucose-6-phosphate (G6PD) function, all molecules that are essential in cellular redox homeostasis. The R001-induced antitumor cell effects were preceded by the early inhibition of the functions of G6PD, TrxR1, and constitutively active STAT3 in TNBC cells, but not of the epidermal growth factor receptor (EGFR) or Janus kinase 2 (Jak2) induction. TNBC, but not normal cells responded to R001 treatment with suppressed GSH levels, but elevated NADPH levels for up to 16 h, and the induction of reactive oxygen species (ROS). The changes in the GSH and NADPH levels reflect a shift in mitochondrial function, evident by a loss of mitochondrial respiration in MDA-MB-436 and MDA-MB-231 cells, but not in the normal human breast epithelial, MCF-10A cells. Furthermore, treatment with R001 of TNBC, but not normal cells induced early pyroptosis and late DNA damage, cell cycle arrest, and apoptosis. In vivo oral administration of 5 mg/kg R001 strongly inhibited the growth of MDA-MB-468 xenografts in mice, which was blocked by co-treatment with the anti-oxidant, *N*-acetyl cysteine. Results altogether indicate that TNBC cells with redox and metabolic adaptations and constitutely active STAT3 are preferrentially more sensitive than normal cells to R001, which induces early oxidative stress and pyroptosis, and late DNA damage, cell cycle arrest, apoptosis, and tumor growth inhibition in vivo.

## Materials and methods

### Cell lines and reagents

The human breast cancer MDA-MB-468 cells were purchased from American Type Culture Collection (ATCC, Manassas, VA) in August 2020 with authentication and mycoplasma-negative report. MDA-MB-231 cells were purchased from the NCI, with the subsequent authentication performed by ATCC and a mycoplasma-negative test performed by IDEXX BioAnalytics (Westbrook, ME) all in Dec 2019. The human breast cancer, MCF-7, normal human breast epithelial MCF-10A, pancreatic cancer line, Panc-1, prostate cancer line, DU145, and non-small cell lung cancer line, A549 have been previously reported [11, 24–27]. Panc-1 cells were authenticated by ATCC on December 11, 2015. Human breast cancer, MDA-MB-436 line was a kind gift from Dr. Xiaojiang Cui of Cedars-Sinai Medical Center, Los Angeles, CA. MCF-10A tested negative for mycoplasma in December 2019 by IDEXX BioAnalytics. HCC1937 cell line was purchased from ATCC in January 2021, with positive authentication and a negative mycoplasma test. These cells were grown in Dulbecco’s modified Eagle’s medium (DMEM) containing 10% heat-inactivated fetal bovine serum (FBS). Human brain microvascular endothelial cells (HBMEC) line was purchased from ScienCell Research Laboratories (Carlsbad, CA). This line was cultured in Clonetics EBM-2 medium. All primary antibodies used were purchased from Cell Signaling Technology (Danvers, MA), except GAPDH, G6PD, and TrxR1 from Santa Cruz Biotechnology (Dallas, TX), Cyclin B1 from Novus Biologicals (Littleton, CO), and Cyclin D1 from Abcam (Waltham, MA). Hydrogen peroxide, *N*-acetyl cysteine (NAC), and glutathione (GSH) were purchased from Sigma-Aldrich (St. Louis, MO), while buthionine sulfoximine (BSO) was purchased from EMD MILLIPORE (Burlington, MA).

### Transfection and siRNA knockdown

The ON-TARGETplus Human STAT3, G6PD, and TrxR1 siRNA were purchased in a SMARTpool format, containing a mixture of 4 siRNA in each pool (Horizon Discovery/Perkin Elmer, Waltham, MA). The control siRNA consisting of a scrambled sequence was purchased from Santa Cruz Biotechnology. The siRNAs were transfected into cells using Lipofectamine RNAiMAX Reagent (Thermo Fisher Scientific, Waltham, MA) according to the manufacturer’s instructions.

### Cell proliferation assay

CyQuant cell proliferation assay (Invitrogen/Life Technologies Corp, Carlsbad, CA) was performed as previously reported [11] and following the manufacturer’s instructions. Relative number of viable cells was normalized to the DMSO-treated control cells.

### Trypan blue exclusion-phase contrast microscopy cell counting

Cells in 6-well plates were transfected with pooled specific siRNA targeting TrxR1 or G6PD or with scrambled siRNA (Scr) and allowed to culture for 72 h. Subsequently, cells were harvested and the viable cells were counted by trypan blue exclusion/phase-contrast microscopy.

### Phase-contrast microscopy for cell morphology

Cells in culture in 6-well plates were untreated or treated once with increasing concentrations of R001 for 24 h. Cells were then imaged under phase-contrast microscopy.

### Clonogenic survival assays

These studies were performed as previously reported [10, 11, 26]. Details are described in Supplementary Info “Materials and Methods” section.

### SDS-PAGE/Western blotting analysis

Whole-cell and tissue lysates were prepared for SDS-PAGE and immunoblotting analysis, as previously reported [11]. Details are described in Supplementary Info “Materials and Methods” section.

### Nuclear extract preparation and gel shift assays

Nuclear extract preparations and electrophoretic mobility shift assay (EMSA) were carried out as previously described [11]. Details are described in Supplementary Info “Materials and Methods” section.

### Flow cytometry for cell cycle and Annexin V binding/apoptotic analysis

For cell cycle analysis, cells were cultured overnight in complete medium. The medium was replaced the next day with fresh serum-free medium and cultured for another 24 h. Subsequently, the medium was replaced with fresh complete medium with or without R001 and the cells were cultured for 24 h. Single cells were harvested and washed with cold PBS, and fixed with fixation buffer (70% ethanol in PBS) overnight at −20 °C. Subsequently, cells were washed two times with PBS, centrifuged at 1500 rpm for 5 min, and the cell pellet was re-suspended in the incubation PI/RNase buffer (BD Biosciences, San Jose, CA) at room temperature for 15 min for staining. DNA content was analyzed by BD FACS LSR II flow cytometry (BD Biosciences), and the cell cycle profile was analyzed by FlowJo V10 software.

For apoptotic analysis, Annexin V/propidium iodide (PI) staining was performed. Briefly, cells in culture and untreated (DMSO, control) or treated with R001 were harvested and stained with FITC-Annexin V (Apoptosis Detection Kit), according to the manufacturer’s instructions (BD Biosciences). The stained cells were washed once with PBS and analyzed by BD LSR II flow cytometer (BD Biosciences) with 488 nm excitation using emission filters appropriate for FITC and R-phycoerythrinand (PE for PI). The data were generated with the FACS DIVA V8.0 software.

### Cellular thioredoxin reductase (TrxR) activity assay

TrxR activity was measured using an assay kit (Abcam) and following the manufacturer’s instructions for determining TrxR activity, with minor modifications. Briefly, tumor tissues or pelleted cells were rapidly homogenized in cold assay buffer with protease inhibitor cocktail (Roche) on ice and centrifuged at 15,000 × *g* for 15 min at 4 °C. The supernatant was collected, and the protein concentration was determined using Bradford assay for each sample. Two sets of samples were tested with or without TrxR inhibitor provided in the kit. The absorbance at 412 nm was measured in a kinetic mode at 25 °C. The activity of the enzyme was calculated and expressed as mU/mg protein.

### Glucose-6-phosphate dehydrogenase (G6PD) activity assay

The activity of G6PD was determined using the G6PD Assay Kit (Sigma-Aldrich) according to the manufacturer’s instructions. Briefly, cells and tumor tissues from treated (with R001) or untreated (DMSO, control), were collected and homogenized in the equivalent volume of ice-cold PBS and centrifuged at 15,000 × *g* for 10 min to remove insoluble materials. Ten microliters (10 µL) of each sample was used in the assay, and all samples and the NADH standards were processed in 96-well plate following the manufacturer’s instructions. The plate was read for absorbance at 450 nm, and the results were processed according to the manufacturer’s instructions. The G6PD activity was expressed as milliunit/mg of total protein in the homogenized supernatant.

### Nicotinamide adenine dinucleotide phosphate (NADP/NADPH) assay

The NADP/NADPH levels in cells and tumor tissues were determined using the NADP/NADPH quantification kit according to the manufacturer’s instructions (Sigma-Aldrich). Details are provided in Supplementary Info “Materials and Methods” section.

### Reduced glutathione (GSH/GSSG) assay

For the detection of reduced glutathione and GSSG levels in tumor tissues and cell lysate, the GSH Assay Kit (Sigma-Aldrich) and GSSG assay kit (Sigma-Aldrich) were used following the manufacturer’s instructions. Details are provided in Supplementary Info, “Materials and Methods” section.

### Measurement of cellular reactive oxygen species (ROS) and hydrogen peroxide (H_2_O_2_) levels

To determine the cellular ROS levels in MDA-MB-231 and MDA-MB-468 cells and the normal human breast epithelial MCF-10A cells, 2′,7′-dichlorodihydrofluorescein diacetate (DCFDA) was used in the assay. Cells were seeded at 20,000 cells/well in black 96-well flat clear bottom tissue-culture plates. After overnight culture at 37 °C, cells were incubated with DCFDA (25 µM) in Hanks Balanced Salt Solution (HBSS, ThermoFisher Scientific, Waltham, MA) at 37 °C for 45 min. The cells in culture were subsequently washed with PBS and untreated (DMSO, control) or treated with R001 or other compounds at different concentrations for different times. Fluorescence signal was measured at 485/535 nm.

To measure cellular ROS levels in cells, a CellROX Deep Red Flow Cytometry Assay Kit (Thermo Fisher Scientific, Waltham, MA) was used. The fluorescence intensity of CellROX Deep Red reflects the ROS levels. Briefly, cells were seeded in a 6-well plate. After incubation overnight, the cells were treated with or without R001 for 16 h. Tert-butyl hydroperoxide (TBHP) was used as a positive control at the concentration of 200 µM for 30 min. Cells were harvested and stained with CellROX Deep Red reagent at a final concentration of 500 nM and incubated for 30 min at 37 °C in the dark. The samples were immediately analyzed by FACS flow cytometry (BD Biosciences) using an APC filter. Approximately 2 × 10^4^ cells were analyzed in each of the samples. Mean fluorescence intensity (MFI) value was analyzed by the FlowJo V10 software.

Mitochondrial superoxide production was monitored by MitoSOX Deep Red staining (#MT14-12, Dojindo, Japan) following the manufacturer’s protocol. Briefly, cells were seeded in 6-well plates (2 × 10^5^ cells per well) and cultured overnight, and then treated with R001 at the indicated time and concentration. Subsequently, the supernatant was removed and the cells were washed two times with HBSS, and the working solutions of MitoSOX red (10 μM) and Hoechst33342 (1 μg/mL) were added, and the cells were further incubated for 30 min at 37 °C in the dark. Thereafter, the cells were washed with HBSS and observed under a fluorescence microscope (KEYENCE, IL).

Hydrogen peroxide (H_2_O_2_) release was assayed using the Amplex Red Hydrogen Peroxide/Peroxidase Assay Kit (Thermo Fisher Scientific) according to the instructions with minor modifications. Briefly, cells were seeded in 96-well plates and allowed to culture overnight. Next day, the culture medium was removed and the cells were washed once with Krebs–Ringer phosphate (KRPG) buffer consisting of 145 mM NaCl, 5.7 mM sodium phosphate, 4.86 mM KCl, 0.54 mM CaCl_2_, 1.22 mM MgSO_4_, 5.5 mM glucose, pH 7.35). Appropriate dilutions of DMSO, R001 or H_2_O_2_ standard in KRPG buffer were added to the cells at 50 μL per well for 0–48 h. Subsequently, the Amplex Red reaction mixture containing 100 μM Amplex Red reagent and 0.2 U/mL horseradish peroxidase in KRPG buffer was added at 50 μL to each well, and the fluorescent intensity was determined using SpectraMax ID5 microplate reader with excitation at 535 nm and emission at 590 nm at the indicated time points. Results were normalized to the fold change of control samples.

### Seahorse assay

Mitochondrial respiration was measured with Agilent Seahorse XFe96/XF Pro according to the manufacturer’s instructions. Details are provided in Supplementary Info “Materials and Methods” section.

### Comet assay for DNA damage

Cells in culture were untreated (DMSO, control) or treated with R001 at the indicated concentrations for 24 h, or in the case of H_2_O_2_ (400 µM), cells were treated for 1 h. DNA damage was measured by the OxiSelect Comet Assay Kit (Cell Biolabs Inc., San Diego, CA) according to the manufacturer’s instructions with all operations conducted under dimmed light to prevent the occurrence of additional DNA damage. Details are provided in Supplementary Info, “Materials and Methods” section.

### 3D spheroid assay

Studies were performed on MDA-MB-231 cells (2000 cells/well) seeded in ultra-low attachment 96-well round bottom plates. Details are provided in Supplementary Info, “Materials and Methods” section.

### Scratch assay for migration

This assay was performed as previously reported [11]. Details are provided in Supplementary Info, “Materials and Methods” section.

### Immunohistochemistry (IHC)

IHC was performed on the tumor tissues through Cedars Sinai Biobank and Research Pathology Resource core facility on a Discovery Ultra staining system (Roche Tissue Diagnostics, Ventana Medical Systems, Inc.). Details are provided in Supplementary Info “Materials and Methods” section.

### Mice and in vivo tumor studies

Mice were housed in specific pathogen-free conditions in the animal facility. All animal experiments were conducted in accordance with the recommendations in the Guide for the Care and Use of Laboratory Animals on a protocol approved by the Institutional Animal Care and Use Committee (IACUC). Five-week-old female athymic nude mice were purchased from Envigo (Indianapolis, IN) and maintained in the institutional animal facilities approved by the American Association for Accreditation of Laboratory Animal Care. Subcutaneous xenograft studies were performed as previously reported [11, 26]. Mice were injected subcutaneously in the right flank area with 7.3 × 10^6^ MDA-MB-468 cells in 100 μL PBS. When an average tumor volume of 100 mm^3^ was established, tumor-bearing mice were grouped so that the mean tumor sizes in all groups were nearly identical. Mice were administered R001 (5 mg/kg, oral gavage, 100 μL PBS with 5% DMSO) every day, 5 times per week for 75 days. For the R001 combination with NAC treatment, NAC was orally given alone (120 mg/kg) [28], or combined with R001 (5 mg/kg) every day, 5 times per week for 60 days. Animals were monitored daily, tumor size was measured with calipers and body weight was taken every 3–4 days. Tumor volumes were calculated according to the formula *V* = 0.52*a*^2^*b*, where *a* is the smallest superficial diameter and *b* is the largest superficial diameter. For each treatment group, the tumor volumes for each set of measurements were statistically analyzed in comparison with the control (vehicle-treated) group using Student’s *t* test.

### Statistical analysis

Statistical analysis was performed using Student’s *t* test. The significance of differences between groups was determined at *p < 0.05, **p < 0.01, and ***p < 0.001. Sample size is stated for each data. No statistical methods were used to predetermine the sample size. There were no animal exclusion criteria and no animals were excluded. No blinding was done.

## Results

### Inhibition of viable cells, anchorage-dependent and independent growth, and migration of triple-negative breast cancer cells

**Fig. 1:**
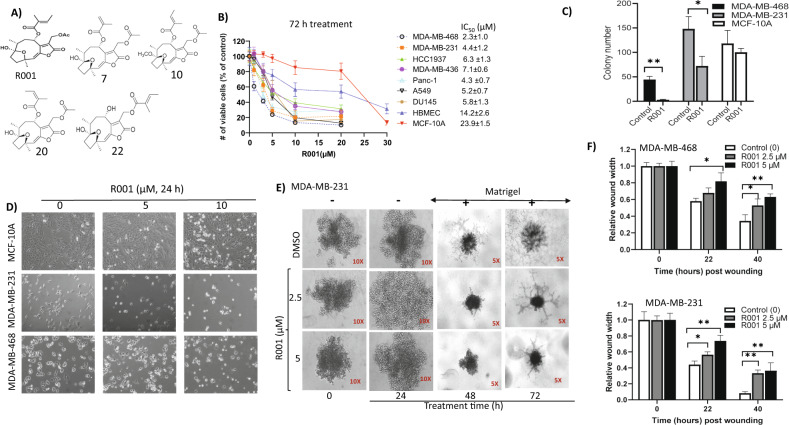
R001 inhibits viable cell numbers, anchorage-dependent and independent growth, and migration and alters the morphology of triple-negative breast cancer cells. **A** Chemical structures of R001, compounds 7, 10, 20, and 22; **B** effects of R001 on the viable cell numbers of the indicated cell lines growing in culture, treated with 0–30 µM R001 for 72 h and subjected to CyQuant cell proliferation assay. Insert IC_50_ values; *n* = 6; **C** single-cell cultures of MDA-MB-468, MDA-MB-231, and MCF-10A cells treated once with 0–1 μM R001 and allowed to grow until large colonies were visible, which were stained with crystal violet, counted and plotted; *n* = 3; **D** phase-contrast microscopic images of the indicated cells growing in culture and untreated or treated with 0–10 µM R001 for 24 h; **E** images taken at 5X and 10X magnifications of MDA-MB-231 cells growing as 3D spheroids formed over a 48-h duration, which were untreated (DMSO) or treated once with 2.5 or 5 μM R001 for 24 h prior to (−) the addition of matrigel (+), and allowed to grow for up to 72 h post treatment and imaged at 24 h intervals; and **F** cultured MDA-MB-468 and MDA-MB-231 cells, which were wounded, treated once with 0–5 µM R001 and allowed to migrate to the denuded area over 22–40 h; *n* = 3. The relative wound width was measured and plotted against treatment time. Control lane (0, –) represents cells treated with 0.1% DMSO. Values, mean ± SD. Data are representative of 2–3 independent determinations. *P < 0.05 and **P < 0.01.

R001 (Fig. 1A) was extracted from the *Vernonia cinerea* plant and characterized as previously reported [23, 29]. R001 was previously extracted as compound 6 together with other hirsutinolides, including 7, 10, 20, and 22 (Fig. 1A) [23]. Details of the extraction and characterization are provided under Supplementary Info “Materials and Methods” section. Purified R001 was dissolved in 100% DMSO for a stock solution of 50 mM (stored at −20 °C), which was freshly diluted down to a series of working solutions with culture medium or phosphate-buffered saline (PBS) and used to achieve the final concentrations as noted in the manuscript. In each study, the final DMSO concentration was no more than 0.1%. The vehicle alone represented 0.1% DMSO in medium or PBS. We evaluated R001 against multiple human cancer lines, including breast cancer MDA-MB-231, MDA-MB-468, MDA-MB-436, MCF-7, and HCC1937, non-small cell lung cancer, A549, pancreatic cancer, Panc-1, and prostate cancer, DU145 cells, and against normal human breast epithelial cells, MCF-10A, and normal human brain microvascular endothelial cells (HBMEC). In CyQuant cell proliferation assay [23, 26], 72-h treatment with R001 dose-dependently suppressed the viable cell numbers of the human cancer lines, MDA-MB-468, MDA-MB-231, Panc-1, A549, DU145, HCC1937, and MDA-MB-436 cells with IC_50_ values of 2.3, 4.4, 4.3, 5.2, 5.8, 6.3, and 7.1 µM, respectively, compared to much weaker effects on the normal human breast epithelial cells, MCF-10A or HBMEC, with IC_50_ of 23.9 or 14.2 µM, respectively (Fig. 1B, IC_50_ insert). One-time treatment with 0–1 µM R001 of single-cell cultures of TNBC cells led to strong inhibition of the colony formation, compared to the minimal effects on the colony formation of MCF-10A cells (Fig. 1C and Supplementary Fig. S1A). Phase-contrast microscopy shows completely altered morphology, including rounding up and decreased cell numbers for the TNBC cells, MDA-MB-231 and MDA-MB-468 following 24-h treatment with R001, compared to minimal effects on similarly treated normal human breast epithelial MCF-10A cells (Fig. 1D). In anchorage-independent growth in 3D matrigel assay, one time treatment with 2.5 or 5 µM R001 led to the degeneration of the mass of MDA-MB-231 cells at 24 h prior to the addition of matrigel (Fig. 1E, 24 h) and following the addition of the matrigel, wherein the treatment further suppressed the colony sizes and the numbers and networks of projections that extended from the colonies at 48–72 h post treatment (Fig. 1E, 48, 72 h). Moreover, scratch assay shows that breast cancer cells treated for short time, 22 h with R001 at the IC_50_ concentrations, 2.5 or 5 µM suppressed the migration of cells into the denuded area at 22 h post treatment (Fig. 1F and Supplementary Fig. S1B). These results together indicate that R001 preferentially inhibits human TNBC cell growth, survival, and migration.

### R001 inhibits the functions of STAT3, glucose-6-phosphate dehydrogenase, and thioredoxin reductase in triple-negative breast cancer cells

**Fig. 2:**
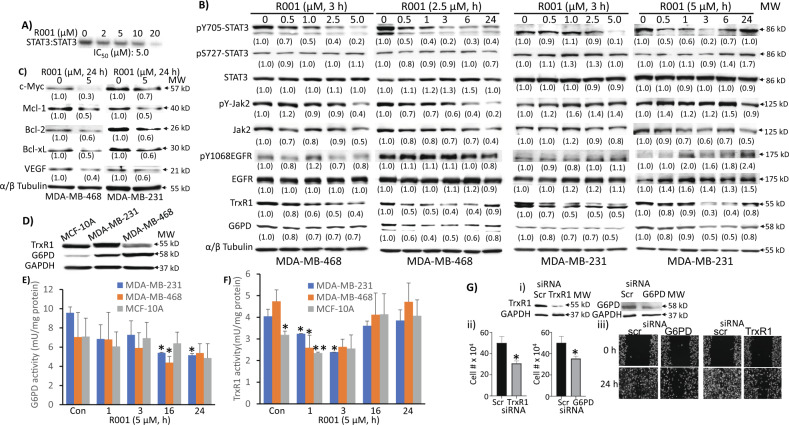
Inhibitory effects of R001 on STAT3 signaling and the expression and activities of glucose-6-phosphate dehydrogenase and thioredoxin reductase, and the effects of G6PD or TrxR1 knockdown on cell growth and migration in breast cancer models. **A** Nuclear extracts of equal total protein containing activated STAT3 prepared from NIH3T3/v-Src fibroblasts were pre-incubated with 0–20 µM R001 for 30 min at room temperature prior to incubating with the radiolabeled hSIE probe that binds STAT3 and performing EMSA analysis; bands corresponding to STAT3:DNA complexes in gel were quantified using ImageJ, plotted against concentration from which the IC_50_ value was derived; **B**–**D** immunoblotting analysis of whole-cell lysates of equal total protein prepared from the MDA-MB-231, MDA-MB-468, or MCF-10A cells **B** treated with or without R001 at 0.5–5 µM for 3 h or with a single concentration, 2.5 µM or 5 µM for 0–24 h, or **C** treated with or without 5 µM for 24 h, or **D** untreated and probing for pY705-STAT3, STAT3, pS727-STAT3, pY-Jak2, Jak2, pY1068EGFR, EGFR, TrxR1, c-Myc, Mcl-1, Bcl-2, Bcl-xL, VEGF, G6PD, tubulin, or GAPDH; protein bands were scanned and quantified using ImageJ and expressed as a fraction of control, which are presented in parentheses; **E**, **F** MCF-10A, MDA-MB-231 and MDA-MB-468 lines in culture were untreated (Con) or treated once with 5 µM R001 for the indicated times and cells were processed and assayed for **E** G6PD or **F** TrxR1 activity, which is plotted against treatment time; *n* = 3 (**G**) MDA-MB-231 cells transfected for 24 h with scrambled (scr) or pooled specific siRNA to knockdown (i) G6PD or TrxR1, and the effects on (ii) viable cell numbers or (iii) cell migration into denuded area; *n* = 3. Positions of proteins or DNA-bound STAT3 in gel are labeled; control lane (0, Con, scr) represents whole-cell lysates prepared from 0.1% DMSO-treated cells, nuclear extracts pre-treated with 10% DMSO, or cells transfected with scrambled siRNA. Values, mean ± S.D. Data are representative of 2–3 independent determinations. *p < 0.05 and **p < 0.01; MW molecular weight.

The hirsutinolides directly inhibited in vitro STAT3 DNA-binding activity in electrophoretic mobility shift assay (EMSA) [23]. Nuclear extracts from the v-Src-transformed mouse fibroblasts (NIH3T3/v-Src) containing activated STAT3:STAT3 dimers [30, 31] were first incubated with increasing concentration of R001 for 30 min at room temperature prior to incubating with the radiolabeled high-affinity *sis*-inducible element (hSIE) probe that binds STAT3 and performing EMSA analysis in vitro [10, 11, 25, 32]. Results show STAT3:STAT3 DNA-binding activity was suppressed by R001, with IC_50_ of 5 µM (Fig. 2A). To further investigate the effects of R001 on intracellular STAT3 signaling, we next treated the TNBC cells, MDA-MB-468 and MDA-MB-231, which harbor constitutively active STAT3 with increasing concentrations of R001 for 3 h or a single concentration, 2.5 or 5 µM for up to 24 h, and prepared whole-cell lysates for immunoblotting analysis. Results showed the inhibition of STAT3 Tyr phosphorylation by R001, which occurred in time- and dose-dependent manner (Fig. 2B, pY705-STAT3), while phospho-Ser-STAT3 (pS727-STAT3), pY1068EGFR, and pY-Jak2 were largely unaffected (Fig. 2B, pS727-STAT3, pY1068EGFR and pY-Jak2). Phospho-Tyr STAT3 is inhibited by R001 at 0.5 µM and as early as 30 min post treatment (Fig. 2B). However, we note that in MDA-MB-231 cells, the pY-STAT3 appears to bounce back at 24 h. Moreover, consistent with the inhibition of constitutive STAT3 functions, treatment with the R001 attenuated the expression of STAT3 downstream target genes, including c-Myc, Mcl-1, Bcl-2, Bcl-xL, and vascular endothelial growth factor (VEGF) in MDA-MB-468 and MDA-MB-231 cells (Fig. 2C). Uncropped Western blot data are presented as part of Supplementary Files, Original Uncropped Western blots.

Our previous studies showed that hirsutinolides inhibit the expression of G6PD and TrxR1 in human glioblastoma models [23]. We extended the studies to examine TNBC cells. Immunoblotting analysis revealed higher expression of G6PD in MDA-MB-231 and MDA-MB-468 cells than in normal human breast epithelial MCF-10A cells, while TrxR1 expression is higher in MDA-MB-231, but lower in MDA-MB-468 cells compared to the normal cells (Fig. 2D). We next conducted both time-course and dose-response studies for the effects of R001 on both G6PD and TrxR1 functions in the breast cancer cells. R001 treatment suppressed both G6PD and TrxR1 levels, changes which occurred as early as 30 min after R001 treatment (Fig. 2B, G6PD, TrxR1). The inhibition of G6PD expression appears gradual and progresses with time. We next asked the question whether the treatment with R001 alters the enzymatic activities of G6PD and TrxR1. For this, we prepared whole-cell lysates from the TNBC and normal breast epithelial cells in culture which had been treated once with R001 for 1–24 h and assayed for G6PD and TrxR1 activities using commercially available kits. Treatment of cells with 5 µM R001 for 1–24 h attenuated the activities of both TrxR1 and G6PD. Compared to the normal MCF-10A cells, the R001-treated MDA-MB-231 and MDA-MB-468 cells showed significant changes in G6PD at 16–24 h (Fig. 2E) and in TrxR1 activities at 1–3 h post treatment (Fig. 2F). The R001-induced change in G6PD activity was more gradual and sustained up to 24 h (Fig. 2E), while TrxR1 inhibition was more rapid (Fig. 2F). On the other hand, the TrxR1 activity appeared to rebound to the control, untreated levels at 16–24 h following R001 treatment, (Fig. 2F), consistent with the return of the protein levels to the control in both TNBC cell lines at a later time point (Fig. 2B, TrxR1, 24 h), while an apparent change in TrxR1 activity in the normal MCF-10A line at 1 h post-treatment was not sustained (Fig. 2F). The rebound of TrxR1 activity is likely compensatory feedback mechanisms, though it is not sufficient for the recovery of the tumor cells.

Altogether, the results indicate that R001 suppresses both the expression and activities of G6PD and TrxR1 in TNBC cells. The suppression of their functions contributes to the R001-induced loss of viable cells. To confirm this, we used siRNA to knockdown both of these genes and examined the effects on TNBC cell growth and migration. Trypan blue exclusion/phase-contrast microscopy showed that siRNA knockdown of TrxR1 or G6PD alone (Fig. 2Gi) suppressed growth of MDA-MB-231 cells (Fig. 2Gii), and similarly, the knockdown of either target alone (Fig. 2Gi) had moderate effects on cell migration in scratch assay (Fig. 2Giii). We conclude that R001 induces an early inhibition of STAT3, G6PD and TrxR1 functions in TNBC cells. The inhibition of STAT3 functions is well established to suppress tumor cell viability, growth, survival and migration [8, 33–35]. The suppression of both G6PD and TrxR1 functions contributes to R001-induced loss of TNBC viable cell numbers.

### R001 treatment specifically modulates NADPH and GSH levels, and mitochondrial respiration in triple-negative breast cancer cells

**Fig. 3:**
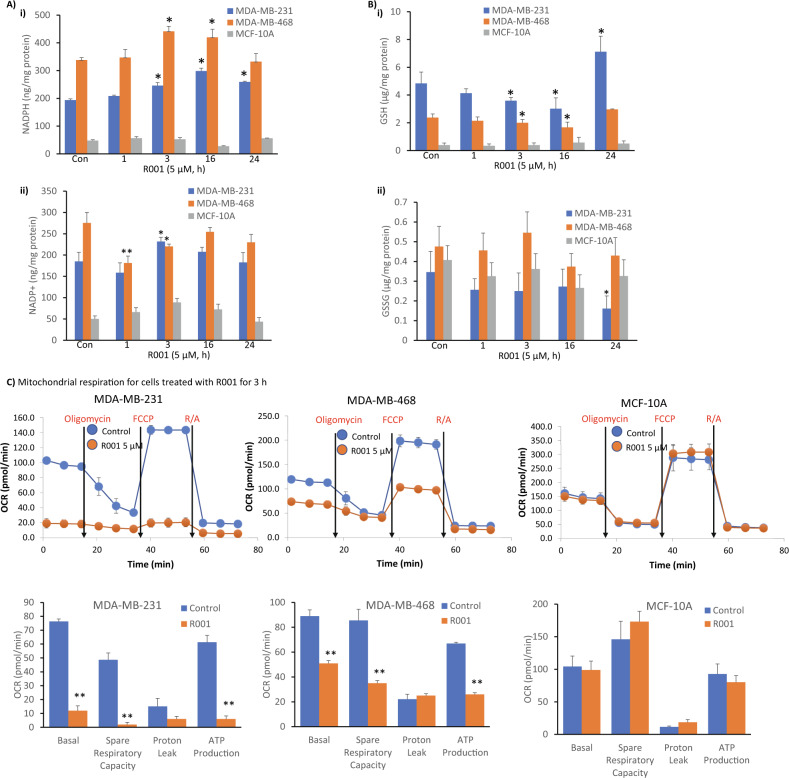

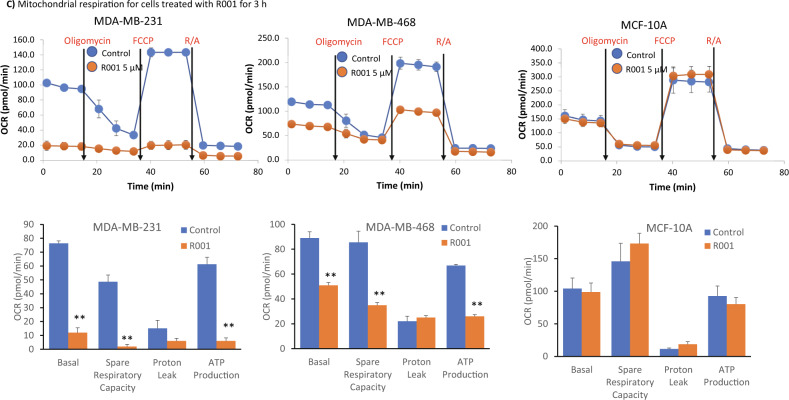
Effects of R001 on the cellular production of NADPH and GSH and mitochondrial functions. **A**, **B** Breast cancer, MDA-MB-231 and MDA-MB-468 cells or normal human breast epithelial, MCF-10A line in culture were untreated (Con) or treated once with 5 µM R001 for the indicated times and cell lysates were prepared and assayed for **A** NADPH (i) and NADP^+^(ii), or **B** GSH (i) and GSSG (ii), which are plotted against treatment time; *n* = 3; and **C** Seahorse analysis for mitochondrial respiration performed on live MDA-MB-231, MDA-MB-468, or MCF-10A lines untreated or treated with 5 µM R001 for 3 h and measured as cellular oxygen consumption rate (OCR) at basal condition and after injection of optimal concentrations of oligomycin, carbonyl cyanide-p-trifluoromethoxy-phenyl-hydrazone (FCCP), and Rotenone plus Antimycin A (R/A), and plotted as line tracing (upper) and bar graph (lower) showing the different phases. Control lane (Control, Con, 0) represents 0.1% DMSO-treated cells; *n* = 3. Values, mean ± S.D. *p < 0.05 and **p < 0.01.

The biochemical functions of G6PD lead to the generation of NADPH, which provides a reducing equivalent for anabolic reactions and is also required for cellular redox balance, including maintaining cellular GSH levels [36], an anti-oxidant. Dysregulation in the GSH homeostasis has been associated with tumor progression and therapy resistance [2]. On the other hand, the TrxR1-thioredoxin antioxidant system utilizes NADPH [37]. Given the inhibitory activities of R001 on both G6PD and TrxR1 functions, we investigated the effects of the natural product on cellular NADPH and GSH levels using commercially available assay kits. We show that both NADPH and NADP^+^ levels in the TNBC, MDA-MB-231 and MDA-MB-468 cells are significantly higher than in the normal human breast epithelial, MCF-10A cells (Fig. 3Ai). The NADPH levels in the tumor cells steadily increased with time following treatment with 5 µM R001, plateaued at 16 h, and returned to near baseline levels at 24 h (Fig. 3Ai). The NADP^+^ levels showed a brief decrease and then returned to baseline in the tumor cells (Fig. 3Aii). By contrast, the NAPD^+^ and NADPH levels in the normal MCF-10A cells, did not change following a similar treatment with R001, except for NADPH decrease at 16 h and NAPD^+^ increase at 3 h that were modest (Fig. 3A, MCF-10A).

For GSH, we observed that its levels are significantly higher in the TNBC cells than in the normal MCF-10A cells (Fig. 3Bi). Importantly, the treatment of TNBC cells with R001 led to time-dependent suppression of GSH levels over a 16-h period in both MDA-MB-231 and MDA-MB-468 cells, with the levels recovering at 24 h (Fig. 3Bi). By contrast, the GSH levels were unchanged in the normal MCF-10A cells following similar treatment with R001 (Fig. 3Bi). The baseline levels of oxidized GSSG were nearly identical in the TNBC and normal cells, with no significant changes induced by R001 (Fig. 3Bii). Therefore, the effects of R001 lead to specific regulation of both the NADPH and GSH levels in TNBC cells but not in normal cells. The background levels of NADPH are higher, associated with lower background GSH levels in MDA-MB-468 cells, compared to the relatively lower background NADPH levels and higher GSH levels in MDA-MB-231 cells (Fig. 3Ai, Bi, Con).

Given the roles of 6-phosphogluconate dehydrogenase (6PGD), malic enzymes (ME 1 and ME 3), and methylenetetrahydrofolate dehydrogenases 1 and 2 (MTHFD1 and MTHFD2) in contributing to the NADPH pool, we investigated the effects of R001 on the expression of these proteins. Immunoblotting analysis shows only small increases in 6PGD and ME 2 levels at 16 h post treatment, no changes in the MTHFD1 and MTHFD2, and a strong, but momentary increase in ME 3 levels that was observed only at 3 h post treatment (Supplementary Fig. S2).

With the changes in NADPH and GSH, we asked whether there is any shift in mitochondrial functions by performing Seahorse studies for mitochondrial respiration. Cells were treated with or without R001 for 3 h. Results showed dramatic alterations of the profile of mitochondrial respiration in the two TNBC cells, MDA-MB-231 and MDA-MB-468 cells treated with R001, while similarly treated normal human breast epithelial MCF-10A cells showed no changes (Fig. 3C). Specifically, R001 induced nearly a complete shut down of mitochondrial respiration in MDA-MB-231 cells (Fig. 3C, MDA-MB-231), while the changes in R001-treated MDA-MB-468 were strong though not to the same extent (Fig. 3C, MDA-MB-468). The differences between the two TNBC lines in this context may be due to the higher baseline NADP^+^/NADPH in MDA-MB-468 than in MDA-MB-231 cells (Fig. 3A). These results show that R001 potently alters mitochondrial respiration selectively in TNBC, but not normal cells.

### R001 treatment promotes ROS induction in triple-negative breast cancer cells

**Fig. 4:**
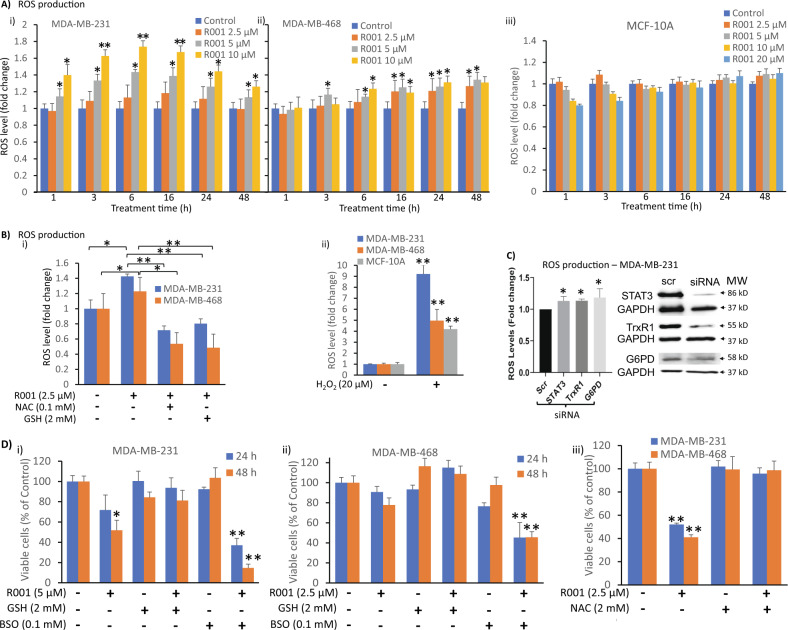
R001 treatment of tumor cells induces ROS production. **A**, **B** MDA-MB-231, MDA-MB-468, or MCF-10A lines were untreated or treated with **A** increasing concentration of R001 alone for the indicated times or **B**i 2.5 µM R001, with or without the anti-oxidant *N*-acetyl cysteine (NAC) or GSH for 24 h, or **B**ii 20 µM hydrogen peroxide (H_2_O_2_) for 24 h, and samples were prepared and assayed for reactive oxygen species (ROS) levels, which are plotted against the treatment duration or the conditions of treatment; *n* = 3; **C** MDA-MB-231 cells were transfected with scrambled (scr) or pooled specific siRNA targeting STAT3, G6PD, or TrxR1 (right), and samples were prepared and assayed for ROS levels, which are plotted (left); *n* = 3; **D** MDA-MB-231 and MDA-MB-468 lines in culture were untreated or treated with 5 µM R001, with or without (i and ii) 2 mM GSH and 0.1 mM buthionine sulfoximine (BSO), or (iii) 2 mM NAC for 24 or 48 h and subjected to CyQuant cell proliferation for viable cell numbers, which are plotted; *n* = 5. Positions of proteins in gel are shown; control lane (Con, 0, −, scr) represents 0.1% DMSO-treated cells or whole-cell lysate prepared from cells transfected with scrambled siRNA. Values, mean ± S.D. Data are representative of 2 or 3 independent determinations. *p < 0.05 and **p < 0.01; MW molecular weight.

Both GSH and NADPH are important in maintaining cellular redox homeostasis. Therefore, we next conducted the biochemical assay for the induction of ROS in cells treated with 2.5–10 µM R001 for 0–48 h. ROS levels showed both dose- and time-dependent increases in MDA-MB-231 cells that peaked around 3–16 h following treatment with R001, and a subsequent decline at 24–48 h (Fig. 4Ai). The picture for MDA-MB-468 cells was slightly different, where the R001-mediated induction of ROS levels was slow and modest, progressed with time, and peaked at 16–48 h (Fig. 4Aii), likely due to the higher background levels of NADPH, which is essential to maintaining a balanced redox cellular environment [38]. By contrast, ROS levels were not significantly altered in the normal human breast epithelial, MCF-10A cells (Fig. 4Aiii), suggesting R001 treatment has minimal impact on the mechanisms that would lead to ROS induction in normal cells. Imaging analyses by CellRox Deep Red or MitoSOX Deep Red staining similarly showed ROS induction in R001-treated TNBC, but not normal MCF-10A cells, with TBHP treatment used as positive control (Supplementary Fig. S3). Similarly, hydrogen peroxide (H_2_O_2_) levels were specifically induced in the tumor cells in response to R001 treatment, with the induction occurring later at 16–24 h in both tumor cells, while H_2_O_2_ levels were unchanged in similarly treated normal MCF-10A (Supplementary Fig. S4A). Treatment with H_2_O_2_ non-specifically induced H_2_O_2_ in all the cell lines (Supplementary Fig. S4B).

The results altogether suggest that treatment with R001 induces oxidative stress in TNBC cells. We sought to suppress the oxidative stress induction in TNBC cells by applying generally accepted anti-oxidant agents, including *N*-acetyl cysteine (NAC) [39] or GSH and to determine how the co-treatment with NAC or GSH would affect R001-mediated ROS induction. The R001-induced ROS was attenuated when the TNBC cells were co-treated with R001 and NAC or GSH (Fig. 4Bi). For positive control, treatment with hydrogen peroxide (H_2_O_2_) led to non-specific, robust ROS induction in both the tumor and normal cells (Fig. 4Bii). These results together suggest that the induction of ROS by R001 is one of its major mechanisms to induce antitumor cell responses. To determine the direct link between the modulation of STAT3, G6PD or TrxR1 functions and the induction of ROS, we used siRNA to knockdown each of the three genes and measured ROS levels. The knockdown of any one of the three targets led to an increase in ROS induction (Fig. 4C).

Combined together, our results indicate that the inhibition of STAT3, G6PD and TrxR1 functions by R001 leads to ROS production and oxidative stress, which induce an antitumor cell response in the TNBC models. To test this further, we co-treated TNBC cells with R001 and anti-oxidant agents and assayed for the viable cell numbers. The co-treatment of R001 with NAC or GSH completely reversed the loss of viable cells (Fig. 4Dii and iii, GSH, NAC). By contrast, the co-treatment of R001 with the GSH synthesis inhibitor, buthionine sulfoximine (BSO) augmented the R001-induced loss of viable cells in both MDA-MB-468 and MDA-MB-231 cells, while BSO alone showed weak or no effect on the viable cells (Fig. 4Di and ii, BSO), indicating that the intracellular GSH might counteract the effects of R001. These results together indicate that the ROS induction in response to R001 treatment inhibits the proliferation of TNBC cells and could be blocked by the addition of exogenous anti-oxidant agents. Furthermore, the lack of notable changes in the NADPH, GSH and ROS levels in MCF-10A cells by the R001 treatment indicates that normal cells are resistant to the effects of R001, which is consistent with the weak effects on the proliferation of the normal cells (Fig. 1B, MCF-10A).

### R001 induces early pyroptosis, and late DNA damage and cell cycle disruption in triple-negative breast cancer cells

**Fig. 5:**
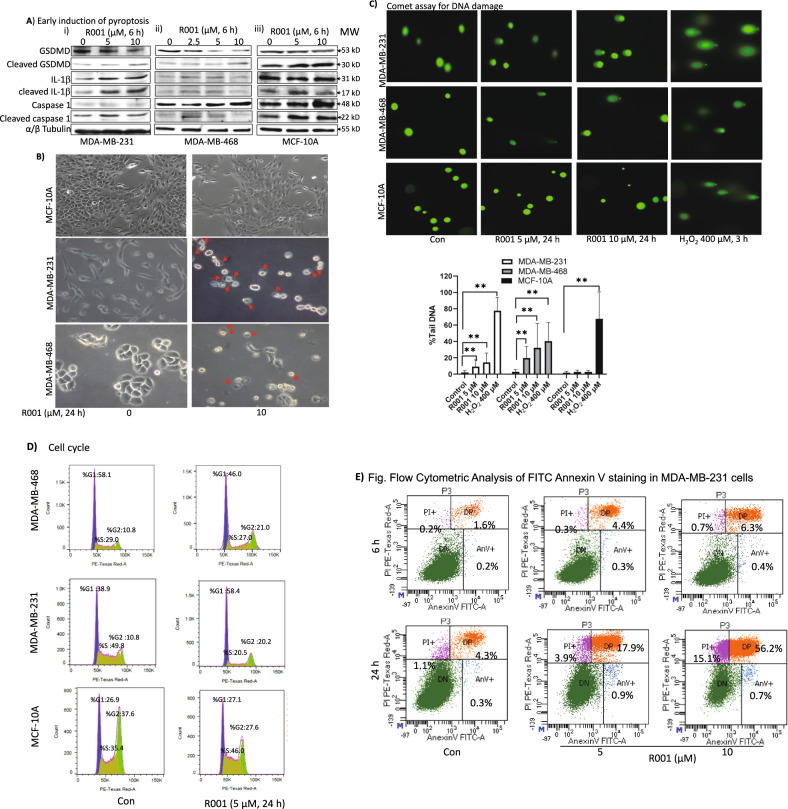

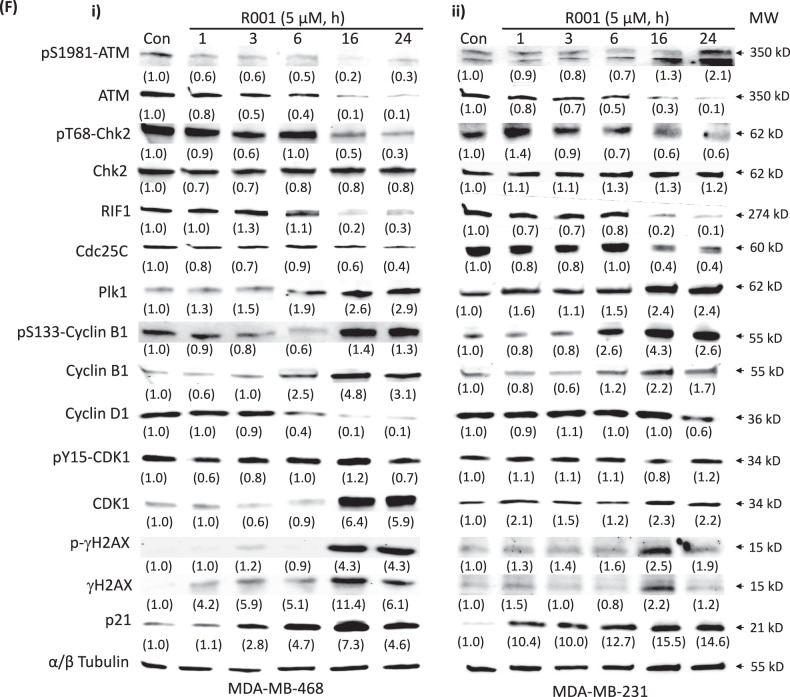
R001 induces early pyroptosis and late DNA damage, altered cell cycle profile, and cell death in triple-negative breast cancer cells. **A**–**E** Triple-negative breast cancer, MDA-MB-231 and MDA-MB-468 cells, or normal human breast epithelial cells, MCF-10A in culture were untreated or treated with 2.5, 5 or 10 µM R001 for 6 or 24 h and **A** whole-cell lysates were prepared and immunoblotted for gasdermin D (GSDMD), cleaved-GSDMD, interleukin-1β (IL-1β), cleaved IL-1β, caspase 1, and tubulin; **B** analyzed for morphology changes under phase-contrast microscopy imaging; red arrows show swollen cells; **C** 24 h subjected to Comet assay. Images were taken under a fluorescence microscope (upper) and quantified for the percent Tail DNA and plotted (lower); *n* = 3; **D** processed, and subjected to flow cytometry analysis for cell cycle progression; **E** cells were processed for Annexin V binding/flow cytometric analysis; and **F** MDA-MB-231 or MDA-MB-468 cells in culture were untreated (Con) or treated with 5 µM R001 for 1–24 h and whole-cell lysates were prepared and subjected to immunoblotting analysis probing for pS1981-ATM, ATM, pT68-Chk2, Chk2, RIF1, Cdc25C, Plk1, pS133-cyclin B1, cyclin B1, cyclin D1, pY15-CDK1, CDK1, p-γH2AX, γH2AX, p21, and tubulin; protein bands were scanned and quantified using ImageJ and represented as a fraction of control, which are shown in parentheses. Positions of proteins in gel are labeled; control lane (con, 0, scr) represents cells treated with 0.1% DMSO or whole-cell lysates prepared from 0.1% DMSO-treated cells. Data are representative of 2 or 3 independent determinations. **p < 0.01; MW molecular weight.

High levels of ROS and oxidative stress are associated with DNA damage [40]. Further, cell death mechanism, including pyroptosis and apoptosis have been causally linked to ROS induction [41, 42]. TNBC cells in culture were treated with R001 and whole-cell lysates were prepared and subjected to immunoblotting analysis to probe for markers of cell death. For both MDA-MB-231 and MDA-MB-468 cells, treatment with R001 led to the cleavage of gasdermin D (GSDMD), caspase-1, and interleukin-1β (IL-1β), which occurred at 6 h (Fig. 5A), indicative of pyroptosis [43]. The results together suggest that the loss of viable cells is in part due to early pyroptosis induced by the high levels of ROS and oxidative stress [41–43]. This is consistent with published reports that ROS induced GSDMD cleavage and pyroptosis in cancer cells [42].

In contrast to the early events, phase-contrast microscopy of the 24-h R001-treated TNBC cells in culture showed altered cellular morphology indicative of fluid-filled cells due to plasma membrane leakage (Fig. 5B, middle to lower panels), which was confirmed by PI staining (Supplementary Fig. S5). We surmise that the prolonged, 24-h treatment of TNBC cells with R001 causes the cells to lose their membrane integrity. We next analyzed for DNA damage using Comet assay [44]. Compared to the untreated control, microscopy analysis of the migration of DNA for the samples prepared from MDA-MB-231 and MDA-MB-468 cells treated with 5 or 10 µM R001 for 24 h showed a comet appearance, with a distinguishable head containing intact DNA and a tail (Fig. 5C, upper, R001), which were quantified (Fig. 5C, lower, R001), indicative of DNA damage. By contrast, the samples from similarly treated normal cells, MCF-10A showed little evidence of DNA damage (Fig. 5C, MCF-10A). Hydrogen peroxide (H_2_O_2_)-treated cells served as a positive control.

With the observation of the DNA damage, we proceeded to determine the impact on cell cycle progression. MDA-MB-231 and MDA-MB-468 cells treated with 5 µM R001 for 24 h, processed and analyzed by flow cytometry for cell cycle progression showed an altered cell cycle profile, with the accumulation of cells at both G1 (58.4 vs 38.9) and in G2 (20.2 vs 10.8) phases, and decreased S-phase population (20.5 vs 49.8) for MDA-MB-231, and largely an accumulation of cells in the G2 phase (21 vs 10.8) for MDA-MB-468, comparing the treated to untreated (DMSO) control cells (Fig. 5D). These results indicate that though both TNBC lines undergo R001-induced cell cycle profile alterations, there are differences in their responses to the natural product. By contrast, similarly treated normal human breast epithelial MCF-10A cells showed no significant changes in the cell cycle profile (Fig. 5D). Annexin V binding with flow cytometric analysis showed no significant induction of early apoptosis at 6 h following R001 treatment, though evidence of extensive cell death of 56.2% occurred at later time (24 h), as indicated by Annexin V and FITC double positive staining (Fig. 5E, 5 or 10 µM, 24 h), while cleavage of poly (ADP-ribose) polymerase (PARP) and caspase 3 at 24–48 h is low to moderate (Supplementary Fig. S6).

We next probed for the regulators of the cell cycle and DNA damage response following treatment of cells with 5 µM R001 for 0–24 h. Consistent with the biological changes, immunoblotting analysis showed the late induction of p-γH2AX and γH2AX [45], suppression of ataxia telangiectasia mutated (ATM) [46] and pS1981-ATM, pT68-Chk2 (checkpoint kinase 2 serine/threonine kinase) [47], rap1 interacting factor (RIF)1 [48], Cdc25C [49], and Cyclin D1, and the induction of p21, CDK1, pS133-Cyclin B1 and Cyclin B1 (Fig. 5Fi and ii). These results together indicate that the prolonged treatment of TNBC cells with R001 promotes DNA damage, leading to cell cycle progression disruption and cell death. The induction of DNA damage is likely caused by the elevated ROS levels [40, 41].

### R001 strongly inhibits growth of subcutaneous human breast cancer xenografts in mice associated with reduced levels of G6PD and Trx1 and phospho-tyr STAT3

**Fig. 6:**
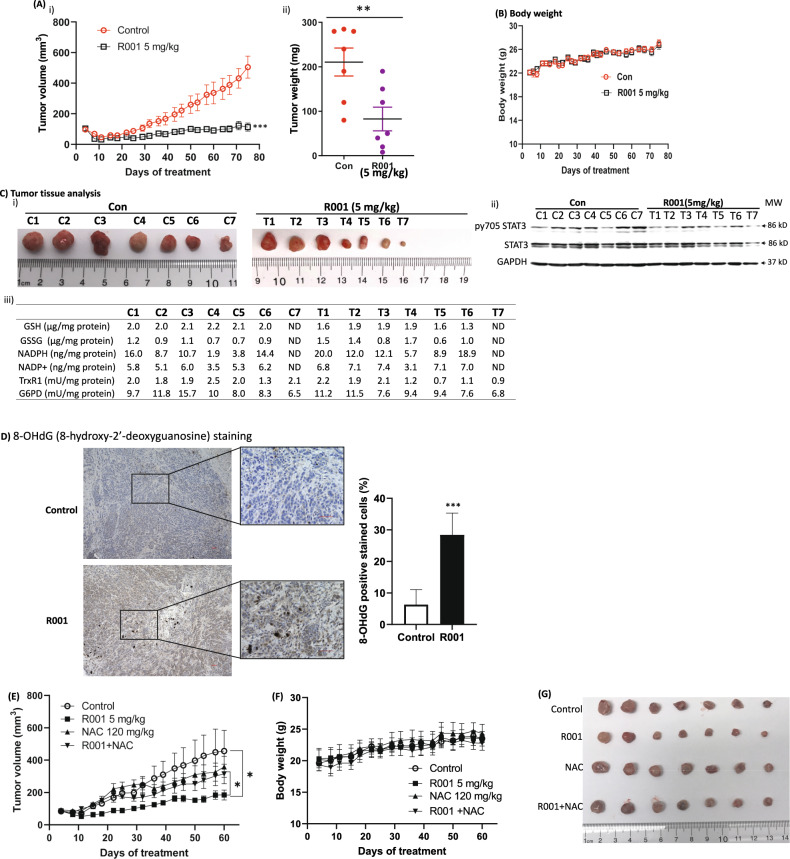
Antitumor effects of R001 against human breast tumor xenografts in vivo. **A** Mice bearing MDA-MB-468 subcutaneous tumor xenografts were administered 5 mg/kg R001 via oral gavage, or vehicle (5% DMSO in PBS) every day, 5 days per week for 75 days. Tumor sizes, measured every 3–4 days, were converted to (A)(i) tumor volumes and plotted against days of treatment, or (ii) tumors were excised at the end of study and weighed and plotted; **B** plot of body weight of tumor-bearing mice versus day of treatment; and **C** control (C1–C7) or residual treated (T1–T7) tumor tissues at the end of study which were imaged and shown (i), or were used to prepare lysates and each subjected to immunoblotting analysis probing for pY705 STAT3, STAT3, and GAPDH (ii), or analyzed for GSH, GSSG, NADPH, and NADP^+^ levels, or TrxR1 and G6PD activities (iii); **D** images (Left panel) of 8-hydroxy-2′-deoxyguanosine (8-OHdG) immunohistochemical staining of tumor tissues from control and R001-treated mice, and a plot of the quantification of three different visual fields of the same slide (right panel); data representative of 3 mice; scale bar = 50 µm; and **E**–**G** mice bearing MDA-MB-468 subcutaneous tumor xenografts were administered 5 mg/kg R001 alone or 120 mg/kg NAC alone via oral gavage, R001 and NAC combined, or vehicle (5% DMSO in PBS) every day, 5 days per week for 60 days. Tumor sizes were measured every 3–4 days, converted to tumor volumes and **E** plotted against days of treatment, or **F** plot of body weight of tumor-bearing mice versus day of treatment; and **G** tumors were excised at the end of study and imaged. Control (Con) represent mice treated with vehicle, 5% DMSO in PBS. Values, mean ± S.E.M., *n* = 7. *p < 0.05, **p < 0.01, and ***p < 0.001; ND not determined, MW molecular weight.

We determined the antitumor effects of R001. Daily oral gavage dosing of 5 mg/kg R001 inhibited in vivo growth in mice of MDA-MB-468 subcutaneous xenografts that harbor aberrantly active STAT3 (Fig. 6A), with no significant changes in body weights (Fig. 6B) or obvious signs of toxicity, such as loss of appetite, decreased activity, or lethargy. Notably, as observed in the in vitro studies, the tissue lysate analyses showed broadly lower activities for G6PD (9.7, 11.8, 15.7, 10.0, 8.0, 8.3 and 6.5 mU/mg protein for control, C1-C7, versus 11.2, 11.5, 7.6, 9.4, 9.4, 7.6 and 6.8 mU/mg protein for treated, T1-T7, respectively) and TrxR1 (2.0, 1.8, 1.9, 2.5, 2.0, 1.3 and 2.1 mU/mg protein for control, C1–C7, versus 2.2, 1.9, 2.1, 1.2, 0.7, 1.1 and 0.9 mU/mg protein for treated T1-T7, respectively) (Fig. 6Ciii), and lower GSH levels (2.0, 2.0, 2.1, 2.2, 2.1 and 2.0 µg/mg protein for control, C1-C6, versus 1.6, 1.9, 1.9, 1.9, 1.6 and 1.3 µg/mg protein for treated T1-T6, respectively) (Fig. 6Ciii). These data points for the GSH and NADPH levels, and the activities of TrxR1 and G6PD are presented as plots for each of the tumor tissues in Supplementary Fig. S7. Similarly, pY705 STAT3 was largely inhibited in the treated T1-T7 compared to the control C1-C7 (Fig. 6Cii). By contrast, NADPH levels were elevated in the residual treated tumor samples compared to non-treated control (Fig. 6Ciii). In another study, similar treatment with R001 of MDA-MB-468 tumor xenografts in mice showed strong inhibition of tumor growth (Fig. 6E, G), with no changes in body weight (Fig. 6F). Consistent with the in vitro data that the co-treatment of R001 with NAC blocks R001-induced ROS production (Fig. 4B) and R001-induced loss of viable cells in treated TNBC cells (Fig. 4Di, iii), co-treatment of R001 and NAC suppressed R001-induced tumor growth inhibition, while NAC alone showed no significant change in tumor growth (Fig. 6E, G, R001 + NAC, NAC). These results indicate the mechanism of antitumor effects of R001 is in part through the induction of oxidative stress. To confirm the induction of oxidative stress in the R001-treated tumor tissues, we conducted 8-hydroxy-2′-deoxyguanosine (8-OHdG) staining of tissues from the MDA-MB-468 tumor xenografts. Compared to the DMSO-treated control, R001-treated tumor tissues showed strong staining for 8-OHdG (Fig. 6D), indicating oxidative stress in the R001-treated tumors.

## Discussion

We report the antitumor activity of R001 against human TNBC in vitro and in vivo, consistent with our previous report of the inhibitory effects against human glioblastoma (GBM) [23]. Herein we investigated the mechanistic underpinning and the significance of the inhibitory activities against STAT3, G6PD, and TrxR1 [23]. The examination of the expression of STAT3, G6PD, and TrxR1 genes in the genomic data from 7569 breast cancer patient samples and 242 non-tumor breast tissues in The Cancer Genome Atlas (TCGA) and Gene Expression Omnibus (GEO) databases shows that the expression of G6PD is significantly higher in TNBC patients compared to normal tissues, and that the patients with the high expression of STAT3, G6PD, or TrxR1 had a poorer overall survival than the patients with low expression of the three genes (Supplementary Fig. S8).

**Fig. 7:**
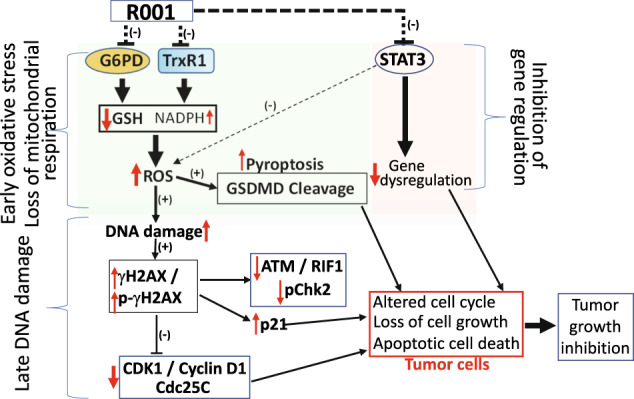
Model of the underlying molecular mechanisms for the antitumor effects of R001 against human TNBC. R001 inhibits the functions of STAT3, G6PD, and TrxR1 early. The inhibition of constitutively active STAT3 leads to the attenuation of the dysregulation of gene expression caused by aberrant STAT3 activity. The modulation of G6PD and TrxR1 collectively alters both GSH levels, which decrease, and NADPH levels, which increase, and these changes are reflective of loss of mitochondrial respiration. These molecular changes contribute to increased ROS production, indicative of oxidative stress, which promotes early pyroptosis (GSDMD cleavage) and a late DNA damage. The consequences of the DNA damage are the induction of the DNA damage response regulators, including p-γH2AX and γH2AX, and altered cell cycle profile, with p21 induction and Cdc25C and Cyclin D1 suppression. These alterations together lead to tumor cell growth inhibition and tumor cell death in the R001-treated TNBC cells, and the inhibition TNBC growth in vivo. (+), induction, (−), inhibition, red up arrow, elevated, red down arrow, suppression.

With the persistent changing gene expression and other cellular events in cancer, the adaptations of tumor cells are necessary for their survival. In that context, the high oxidative burden in the tumor cells likely creates a dependency on the G6PD and TrxR1 functions as part of the adaptation mechanisms for tumor cell survival, given the roles of the two targets in cellular redox events [1, 2]. The dependence on aberrantly active STAT3 to support tumor cell growth and survival is well established [4, 5, 23, 50]. Thus, the present results together highlight the importance of the three targets for the antitumor effects of R001 against TNBC cell growth, cell survival, and tumor growth in a model (Fig. 7) in which STAT3, G6PD, and TrxR1 are targets of the early inhibition by R001. This leads to the suppression of the pro-tumorigenic, STAT3-dependent target genes, and the early reduction in GSH, with an elevation of NADPH levels (Fig. 7). The surge in the NADPH levels in response to R001 treatment could partly be due to the stronger inhibitory effect of the natural product on the TrxR1-thioredoxin anti-oxidant system that consumes NADPH [37], compared to the moderate effect on the G6PD activity, which produces NADPH via the direct metabolism of glucose-6-phosphate and the link to the pentose phosphate pathway [36]. The elevated NADPH presumable is incapable of blunting or reversing the antitumor cell effects of the early oxidative stress induced in the R001-treated TNBC cells. The changes in GSH and NADPH levels reflect a shift in mitochondrial function. Notably, R001-treatment led to a strong or near complete loss of mitochondrial respiration in MDA-MB-436 and MDA-MB-231 cells, respectively, with no noticeable changes in the normal human breast epithelial, MCF-10A cells.

The redox and metabolism adaptations to the high oxidative stress environment in cancer cells [1] are manifested by the higher levels in the TNBC cells of the anti-oxidant molecules, GSH and NADPH, the latter which is a major source of reducing equivalent. Comparing the TNBC and normal cells, our findings suggest that the redox adaptation mechanisms of the tumor cells make them more susceptible to R001 effects, compared to normal cells which have lower GSH and NADPH levels and are less sensitive. Consistent with this, our findings further show that R001 concurrently induce strong alteration of mitochondrial respiration. The early reduction in GSH facilitates the induction of ROS (oxidative stress), which promotes the loss of viable cells via mechanisms that include the early induction of pyroptosis [42, 43] in the TNBC models (Fig. 7). There appears to be differences between the two TNBC cells, MDA-MB-231 and MDA-MB-468, with regard to ROS induction and NADPH production in response to R001 treatment, though both lines are responsive to the natural product in the overall antitumor effects.

The induction of the early oxidative stress events by R001 triggered unrecoverable DNA damage [40, 41] that occurs late following the treatment of the TNBC cells with the natural product. This is evident by the late induction of both p-γH2AX and γH2AX [45], concomitant with the suppression of ATM, pS1981-ATM, RIF1, and pT68-Chk2, all components of the DNA damage response [46–48]. Additional changes induced in response to R001 include the early p21 induction and the downregulation of Cdc25C [49] and Cyclin D1, which collectively perturbed cell cycle progression, including causing cell accumulation at both G1 (58.4 vs 38.9) and G2 (20.2 vs 10.8), with and a decrease in the S-phase population (20.5 vs 49.8) for MDA-MB-231, and largely an accumulation of cells in the G2 phase (21 vs 10.8) for MDA-MB-468, comparing treated to untreated, (DMSO) control. The outcome of these changes are cell death by early pyroptosis and a late apoptosis in the R001-treated TNBC cells (Fig. 7). In contrast to the responses of the tumor cells to R001, normal cells were little affected by the natural product.

The in vivo administration of R001 inhibited growth of subcutaneous human MDA-MB-468 xenografts in mice, with no evidence of toxicity. Consistent with the in vitro data, the antitumor effects are in part due to the induction of oxidative stress, as evident by the detection of high ROS levels in the R001-treated tumor tissues, and further supported by the co-treatment with NAC, which blocked the R001 antitumor response in vivo. Results altogether define R001-mediated mechanisms of antitumor effects against human TNBC models involving the inhibition of STAT3, G6PD and TrxR1 functions. The effects of R001 include the early induction of oxidative stress events in TNBC cells, leading to DNA damage, altered cell cycle profile, and tumor cell death, and tumor growth inhibition in vivo. Results further show that TNBC cells with redox and metabolic adaptation mechanisms are more susceptible than normal cells to the R001-mediated effects.

## Supplementary information

Supplementary Materials

Supplementary Materials

Supplementary Materials

Checklist

Reagents List

## Data Availability

The data generated in this study are available within the article and its Supplementary Data files.
