# Supplementary material for: Natural product preferentially targets redox and metabolic adaptations and aberrantly active STAT3 to inhibit breast tumor growth in vivo
Source: Cell Death Dis. 2022 Dec 6;13(12):1022. doi: 10.1038/s41419-022-05477-2 (PMC9726930; doi:10.1038/s41419-022-05477-2)
Supplement: Supplementary file 1 — Supplementary Materials [file 41419_2022_5477_MOESM1_ESM.docx]

**Natural product preferentially targets redox and metabolic adaptations and aberrantly-active STAT3 to inhibit breast tumor growth**

Yinsong Zhu^1,2^, Peibin Yue^1,2^, Cody F. Dickinson^3^, Justin K. Yang^3^, Kyrstin Datanagan^3^, Ning Zhai^1,2^, Yi Zhang^4^, Gabriella Miklossy^5^, Francisco Lopez-Tapia^1,2^, Marcus A. Tius^3,5^, and James Turkson^1,2,*^

^1^Department of Medicine, Division of Medical Oncology and ^2^Cedars-Sinai Cancer, Cedars-Sinai Medical Center, 8700 Beverly Blvd, Los Angeles, CA, USA 90048, ^5^Department of Chemistry, University of Hawaii, Manoa, 2545 McCarthy Mall, Honolulu, HI, USA 96825, ^4^Biobank and Research Pathology Resource, Academic Affairs and Research Administration, Cedars Sinai Medical Center, 8700 Beverly Boulevard, Los Angeles, CA, USA 90048, ^5^Cancer Biology Program, University of Hawaii Cancer Center, 701 Ilalo St, Honolulu, HI, USA 96813,

Running title: Hirsutinolide targets redox adaptations and STAT3 activity.

*Address correspondence to:

James Turkson, Professor

Department of Medicine

Division of Hematology-Oncology

Cedars-Sinai Cancer

Cedars Sinai Medical Center

8700 Beverly Blvd, Davis 5065

Los Angeles, CA, 90048

Tel. 310-423-6887

Email: [james.turkson@cshs.org](mailto:james.turkson@cshs.org)

**Materials and Methods**

*Source of hirsutinolide-* The hirsutinolide, R001 was derived from the *Vernonia cinerea* plant. The isolation from *V. cinerea*, structural elucidation, and the characterization of the hirsutinolide have been previously reported ^1, 2^. Two improved methods for the extraction of R001 from of the dried plant material have been developed. [Footnote: for full details see the experimental section] The first is a streamlined version of the previously described extraction protocol ^1^. Four kilograms of coarsely ground dried plant material was divided into three approximately equal portions and each was soaked in methanol (4 L) for 24 h. The process was repeated two more times and the methanol was collected each time and combined. After evaporation of the solvent, the crude extract was diluted in 1:1 acetonitrile:water and passed through a C18 silica gel column. The fractions that contained R001 were combined, the acetonitrile evaporated, and the remaining aqueous phase extracted with dichloromethane (5x). Silica gel chromatography followed by several aqueous sodium bicarbonate washes to remove residual fatty acids afforded 160 mg of R001 (*ca.* 40 ppm from dry plant material) in 94% purity as determined by LCMS and ^1^H NMR. [Footnote: The product representing the remaining 6% of the mass was not identified, but on the basis of its molecular weight is likely to be the known C8 2-methacrylate; ^1^H NMR data of R001 matched the spectrum reported in ^3^.

The second method of extraction using supercritical carbon dioxide (sCO_2_) proved to be more convenient and higher yielding. A single (unoptimized) extraction of 2.8 kg of the same batch of dried plant material using sCO_2_ followed by a sCO_2_/ethanol mixture was carried out. One advantage of this procedure is the fractionation and the effective de-fatting that take place in the early stages of the extraction. A total of 314 g of non-polar byproducts were removed from the plant material in this way. Also, the sugars are not extracted to any significant extent even during the last ethanol wash. As a result, in addition to avoiding the use of large volumes of flammable and toxic methanol, this method leads to a much lower mass of extract that is more highly enriched in R001. The final ethanol wash of the plant material containing the majority of R001 was combined with the next to last scCO_2_ extract. Washing the residue thoroughly with acetonitrile into a separate flask followed by evaporation of the solvent gave a thick syrup that was suspended in 1:1 acetonitrile:water. After soaking for 24 h the solids were removed by filtration through Celite. The acetonitrile was removed by evaporation and the remaining aqueous phase was extracted with dichloromethane (5x) and concentrated. R001 was purified through a similar series of operations as described above. Passage through a C18 silica gel column, further purification on normal phase silica gel, and washing with aqueous sodium bicarbonate provided 135 mg and 23 mg of R001 (*ca.* 56 ppm from dry plant material) in two grades of purity, 92% and 90%, respectively. [Footnote: The product representing the remaining 6% of the mass was not identified, but on the basis of its molecular weight is likely to be the known C8 2-methacrylate; ^1^H NMR data of R001 matched the spectrum reported in ^3^.

General methods

The dried plant material was sourced from Thailand and purchased from Lampang Herb Conservation, www.lampangherbs.com. Methanol (MeOH, ACS certified), ethanol (EtOH, ≥ 95%) toluene (PhMe, ACS certified), acetonitrile (MeCN, HPLC grade), dichloromethane (ACS certified), diethyl ether (Et_2_O, ACS certified) was purchased from Fischer Chemical and used without further purification. Column chromatography was performed on C18 silica gel (endcapped, porosity 60 Å, particle size 40-75 μm, pH 6-8, carbon load ~19%, bulk density 0.4 g/mL) and silica gel (200-400 mesh) or premium silica gel (porosity 60 Å, particle size 40–75 µm).^1^H NMR spectra were measured on a Varian Mecury-300 (300 MHz) spectrometer at ambient temperature. Chemical shifts are reported in parts per million (ppm) and are referenced to the solvent (*e.g.,* δ 7.26 for CHCl_3_). Multiplicities are indicated as follows: br (broadened), s (singlet), d (doublet), t (triplet), q (quartet), pent (pentet), sext (sextet), sept (septet), etc. or m (multiplet). Coupling constants (*J*) are reported in Hertz (Hz). High-resolution mass spectra (HRMS) were obtained with an Agilent 1100 quaternary LC system. Supercritical carbon dioxide (sCO_2_) extraction was performed on a single 10 L Green Mill Supercritical SFE Pro by Mr. Jordan Medeiros at Aloha Green Apothecary (Honolulu, HI). Thin layer chromatography (TLC) was performed on glass plates, 250 µm, particle size 5–17 µm, pore size 60 Å. Purity and homogeneity were determined by ^1^H NMR, TLC, and LCMS.

Method A:

In the typical procedure 4 kg of the plant were coarsely ground in a food blender and divided equally between three vessels containing 4 L of reagent grade methanol for 24 h. The extract was decanted through a glass fritted funnel. This process was repeated two more times for a total of three extractions of the bulk plant material. The combined organic extracts were concentrated to give a thick syrup. The syrup was soaked overnight in a 1/1 MeCN/water mixture (approx. 500–600 mL), filtered through Celite using a glass fritted vacuum funnel, and washed thoroughly with 1/1 MeCN/water. After evaporation of the MeCN, the aqueous phase was extracted with dichloromethane (approx. 150–200 mL, 5x), filtered to remove any solids, and concentrated to dryness. The residue was loaded onto a C18 column (approximate height and diameter of C18 silica gel in the column was 11 cm and 6 cm, respectively) as a 1/1 MeCN/water mixture and eluted with 1/1 MeCN/water. Six fractions of approximately 250 mL each were collected and analyzed by LCMS for hirsutinolide. The fractions that contained hirsutinolide were combined and concentrated. The aqueous phase was extracted with dichloromethane (150–200 mL, 5x). The combined organic extracts were washed with brine and dried over anhydrous MgSO_4_. After filtration, the solvent was removed *in vacuo* and the crude oil was dry loaded onto a silica gel column (approximate height and diameter of the silica gel in the column was 18 cm and 2 cm, respectively). A solvent gradient of 0, 5, 10, 15, 20, 25, 30, 35, 40, 45, 80 and100% ethyl acetate in hexane was used to elute the desired compound. Hirsutinolide eluted in the 25% ethyl acetate/hexane fraction. The hirsutinolide was further purified on a second silica gel column (approximate height and diameter of the silica gel in the column was 18 cm and 2 cm, respectively) using a gradient of 0, 5, 10, 15, 20, 25, 30, 35 and 40% diethyl ether in toluene as eluent. The hirsutinolide was still contaminated by fatty acids. The fatty acids were removed by washing a dichloromethane solution (approximately 20 mL) of the mixture with an equal volume of saturated aqueous NaHCO_3_, followed by centrifugation to separate the emulsion, and finally removal of the dichloromethane phase. The dichloromethane phase was again washed with NaHCO_3_, centrifuged, and separated for a total of three times. The combined organic phases were dried with anhydrous MgSO_4_, filtered, and concentrated to give 160 mg of hirsutinolide in 94% purity from the 4 kg of plant material. The ^1^H NMR of R001 matched the previously reported spectrum ^1^.

Method B:

Approximately 2.8 kg of the dried plant material was subjected to sCO_2_ extraction. The extracts were analyzed by LCMS for hirsutinolide. The materials from collections 3 and 4 (see table below) were found to contain hirsutinolide, were combined as acetonitrile solutions in a separate flask (approximately 500–600 mL of acetonitrile was used), and then concentrated to provide a thick syrup. The thick syrup was suspended in a 1:1 acetonitrile:water mixture (*ca.*1 L) and allowed to soak for 24 h. Any insoluble solids were removed by filtration through Celite. The acetonitrile was removed by evaporation and the remaining aqueous phase was extracted with dichloromethane (*ca.* 150–200 mL; 5x) and the organics concentrated. Isolation and purification of the natural product by C18 silica and silica gel was carried out in the same manner as described for Method A. This provided 135 mg and 23 mg of R001 in two grades of purity, 92% and 90%, respectively. The ^1^H NMR of R001 matched the previously reported spectrum ^1^.

| Vessel | T (°C) | P (bar) | Density (g/mL) | Yield |
| --- | --- | --- | --- | --- |
| Extraction^a^ | 52 | 240 | 0.854 | - |
| Collection 1^a^ | 55 | 195 | 0.747 | 1.58 g |
| Collection 2^a^ | 45 | 95 | 0.414 | 73.05 g |
| Collection 3^a^ | 19 | 48 | 0.132 | 239.83 g |
| Collection 4^b^ | - | - | - | - |

a. Settings: HCT = 55 °C, time elapsed = 17.5 h, flow rate = 259.4 g/min.

b. Ethanol cleaning cycle, load extraction vessel with 100 – 500 mL ethanol (≥ 95%), time elapsed = 3 h, flow rate = 350 g/min.

(4*S*,6*R*,7*S*,10*R*,*E*)-3-(acetoxymethyl)-7-hydroxy-6,10-dimethyl-2-oxo-2,4,5,6,7,8,9,10-octahydro-7,10-epoxycyclodeca[*b*]furan-4-yl (*E*)-2-methylbut-2-enoate (**R001**)

^1^H NMR (300 MHz, CDCl_3_) 7.00 (qq, *J* = 7.0, 1.4 Hz, 1H), 6.25 (d, *J* = 7.6 Hz, 1H), 5.87 (s, 1H), 5.09 (d, *J* = 13.0 Hz, 1H), 5.00 (d, *J* = 13.0 Hz, 1H), 2.34 – 2.24 (m, 2H), 2.11 – 2.03 (m, 6H), 1.92 – 1.71 (m, 9H), 1.47 (s, 3H), 2.99 (d, *J* = 6.9 Hz, 3H).

*NMR spectra*

Biology Methods

**Materials and Methods**

*Cell lines and reagents-* The human breast cancer MDA-MB-468 cells were purchased from American Type Culture Collection (ATCC, Manassas, VA) in Aug 2020 with authentication and mycoplasma negative report. MDA-MB-231 cells were purchased from the NCI, with the subsequent authentication performed by ATCC and a mycoplasma negative test performed by IDEXX BioAnalytics (Westbrook, ME) all in Dec 2019. The human breast cancer, MCF-7, normal human breast epithelial MCF-10A, pancreatic cancer line, Panc-1, prostate cancer line, DU145, and non-small cell lung cancer line, A549 have been previously reported ^4, 5, 6, 7, 8^. Panc-1 cells were authenticated by ATCC on December 11, 2015. Human breast cancer, MDA-MB-436 line was a kind gift from Dr. Xiaojiang Cui of Cedars-Sinai Medical Center, Los Angeles, CA. MCF-10A tested negative for mycoplasma in Dec 2019 by IDEXX BioAnalytics. HCC1937 cell line was purchased from ATCC in January 2021, with positive authentication and a negative mycoplasma test. These cells were grown in Dulbecco's modified Eagle's medium (DMEM) containing 10% heat-inactivated fetal bovine serum (FBS). Human brain microvascular endothelial cells (HBMEC) line was purchased from ScienCell Research Laboratories (Carlsbad, CA). This line was cultured in Clonetics EBM-2 medium. All primary antibodies used were purchased from Cell Signaling Technology (Danvers, MA), except GAPDH, G6PD, MTHFD1 and TrxR1 from Santa Cruz Biotechnology (Dallas, TX), 8-OHdG from Bioss Inc (Woburn, MA), Cyclin B1 from Novus Biologicals (Littleton, CO), and Cyclin D1 from Abcam (Waltham, MA). Hydrogen peroxide, *N*-acetyl cysteine (NAC), and glutathione (GSH) were purchased from Sigma-Aldrich (St. Louis, MO), while buthionine sulfoximine (BSO) was purchased from EMD MILLIPORE (Burlington, MA).

*Transfection and siRNA knockdown*- The ON-TARGETplus Human STAT3, G6PD, and TrxR1 siRNA were purchased in a SMARTpool format, containing a mixture of 4 siRNA in each pool (Horizon Discovery/Perkin Elmer, Waltham, MA). The control siRNA consisting of a scrambled sequence was purchased from Santa Cruz Biotechnology. The siRNAs were transfected into cells using Lipofectamine RNAiMAX Reagent (Thermo Fisher Scientific, Waltham, MA) according to the manufacturer’s instructions.

*Cell proliferation assay-* CyQuant cell proliferation assay (Invitrogen/Life Technologies Corp, Carlsbad, CA) was performed as previously reported ^8^ and following the manufacturer’s instructions. Relative number of viable cells was normalized to the DMSO-treated control cells.

*Trypan blue exclusion-phase contrast microscopy cell counting*- Cells in 6-well plates were transfected with pooled specific siRNA targeting TrxR1 or G6PD or with scrambled siRNA (Scr) and allowed to culture for 72 h. Subsequently, cells were harvested and the viable cells were counted by trypan blue exclusion/phase-contrast microscopy.

*Phase-contrast microscopy for cell morphology*- Cells in culture in 6-well plates were untreated or treated once with increasing concentrations of R001 for 24 h. Cells were then imaged under phase-contrast microscopy.

*Clonogenic survival assays-* These studies were performed as previously reported ^6, 8, 9^. Briefly, cells were seeded as single-cell in 6-well plates (250 cells per well), treated once the next day with R001 at the indicated concentrations, and allowed to culture for 2 or 3 weeks when large colonies were visible. Colonies were stained with crystal violet for 4 h and photographed with KwikQuant imager (Kindle Biosciences, Greenwich, CT), and clearly visible colonies were counted.

*SDS-PAGE/Western blotting analysis-* MDA-MB-231 or MDA-MB-468 cells in culture were treated or untreated with different concentrations of R001 for different times. Cultured cells were harvested and whole-cell lysates prepared for SDS-PAGE and immunoblotting analysis, as previously reported ^8^. Tumor tissues were homogenized in cell lysis buffer with protease inhibitor cocktail (Roche) and prepared in the same way as for the cultured cells. Primary antibodies used were anti- STAT3, pY705STAT3, pJak2, Jak2, pY1068EGFR, EGFR, G6PD, TrxR1, c-Myc, Mcl-1, Bcl-2, Bcl-xL, VEGF gasdermin D (GSDMD), cleaved-GSDMD, interleukin-1 (IL-1), caspase 1, caspase 3, poly (ADP-ribose) polymerase (PARP), pS1981-ATM, ATM, pT68-Chk2, Chk2, RIF1, CDC25c, Plk1, pS133-Cyclin B1, Cyclin B1, CyclinD1, pY15-CDK1, p-γ-H2AX, H2AX, p21, ME2, ME3, 6PGD, MTHFD1, MTHFD2, β-tubulin, and GAPDH.

*Nuclear extract preparation and gel shift assays-* Nuclear extract preparations and electrophoretic mobility shift assay (EMSA) were carried out as previously described ^8^. The ^32^P-labeled oligonucleotide probes used were hSIE (high-affinity *sis*-inducible element from the *c-fos* gene, m67 variant (5′-AGCTTCATTTCCCGTAAATCCCTA-3′) that binds STAT3. Nuclear extracts prepared from NIH3T3/vSrc cells containing constitutively-active STAT3 were pre-incubated with increasing concentration of R001 for 30 min at room temperature prior to incubation with the radiolabeled probe for 30 min before subjecting to EMSA analysis.

*Flow cytometry for cell cycle and annexin V binding/apoptotic analysis-* For cell cycle analysis, cells were cultured overnight in complete medium. The medium was replaced the next day with fresh serum-free medium and cultured for another 24 h. Subsequently, the medium was replaced with fresh complete medium with or without R001 and the cells were cultured for 24 h. Single cells were harvested and washed with cold PBS, and fixed with fixation buffer (70% ethanol in PBS) overnight at ‐20 °C. Subsequently, cells were washed two‐times with PBS, centrifuged at 1,500 rpm for 5 min, and the cell pellet was re-suspended in the incubation PI/RNase buffer (BD Biosciences, San Jose, CA) at room temperature for 15 min for staining. DNA content was analyzed by BD FACS LSR II flow cytometry (BD Biosciences), and the cell cycle profile was analyzed by FlowJo V10 software.

For apoptotic analysis, Annexin V/propidium iodide (PI) staining was performed. Briefly, cells in culture and untreated (DMSO, control) or treated with R001 were harvested and stained with FITC-Annexin V (Apoptosis Detection Kit), according to manufacturer’s instructions (BD Biosciences). The stained cells were washed once with PBS and analyzed by BD LSR II flow cytometer (BD Biosciences) with 488 nm excitation using emission filters appropriate for FITC and R-phycoerythrinand (PE for PI). The data were generated with FACS DIVA V8.0 software.

*Cellular thioredoxin reductase (TrxR) activity assay-* TrxR activity was measured using an assay kit (Abcam) and following to the manufacturer’s instructions for determining TrxR activity, with minor modifications. Briefly, tumor tissues or pelleted cells were rapidly homogenized in cold assay buffer with protease inhibitor cocktail (Roche) on ice and centrifuged at 15,000 g for 15 min at 4 ºC. The supernatant was collected, and the protein concentration was determined using Bradford assay for each sample. Two sets of samples were tested with or without TrxR inhibitor provided in the kit. The absorbance at 412 nm was measured in a kinetic mode at 25 ºC. The activity of the enzyme was calculated and expressed as mU/mg protein.

*Glucose-6-phosphate dehydrogenase (G6PD) activity assay*- The activity of G6PD was determined using the G6PD Assay Kit (Sigma-Aldrich) according to the manufacturer’s instructions. Briefly, cells and tumor tissues from treated (with R001) or untreated (DMSO, control), were collected and homogenized in the equivalent volume of ice-cold PBS and centrifuged at 15,000 g for 10 min to remove insoluble materials. Ten microliters (10 µl) of each sample was used in the assay, and all samples and the NADH standards were processed in 96-well plate following the manufacturer’s instructions. The plate was read for absorbance at 450 nm, and the results were processed according to the manufacturer’s instructions. The G6PD activity was expressed as milliunit/mg of total protein in the homogenized supernatant.

*Nicotinamide adenine dinucleotide phosphate (NADP/NADPH) assay*- The NADP/NADPH levels in cells and tumor tissues were determined using the NADP/NADPH quantification kit according to the manufacturer’s instructions (Sigma-Aldrich). Briefly, tumor tissues or cells treated or untreated were washed with cold PBS, and the tissues were homogenized. Cell pellets and homogenized tissues were suspended in the NADP/NADPH extraction buffer on ice according to the manufacturer’s instructions. To remove enzymes that consume NADPH, the extraction supernatant containing NADP/NADPH was deproteinized using 10 kDa cut-off spin filter (Pall Corporation, Port Washington, NY). To decompose NADP^+^ and only detect NADPH, the deproteinized samples were heated at 60 ^o^C for 30 min, allowed to cool, and the cooled samples were centrifuged to remove any precipitates. The total NADP and NADPH levels in the samples were determined based on NADPH standard curve that was generated according to the manufacturer’s instructions. NADP^+^ was calculated by subtracting NADPH from the total NADP. Data were expressed as ng per milligram (mg) of total proteins in the extracted supernatant as determined by Bradford protein assay (Bio Rad).

*Reduced and oxidized glutathione (GSH/GSSG) assay*- For the detection of reduced glutathione levels in tumor tissues and cell lysate, the Reduced GSH Assay Kit (Sigma-Aldrich) was used following the manufacturer’s instructions. Two sets of equally treated tumor tissues or cell suspensions were prepared. One set was rapidly homogenized with 5% sulfosalicylic acid to determine GSH and another set was homogenized with the GSH assay buffer provided in the kit to assay for the protein concentration for each sample. The absorbance at 450 nm was measured in a kinetics mode with time interval of 5 min. For the GSSG levels, the GSH/GSSG assay kit was used following the manufacturer’s instructions. Scavenger was added to the cell lysate to clear all existing GSH, and the optical density of each sample was measured at 412 nm. The amounts of GSH in the samples were adjusted by the corresponding protein concentration and expressed as µg/mg protein.

*Measurement of cellular reactive oxygen species (ROS) and Hydrogen peroxide (H_2_O_2_) levels*- To determine the cellular ROS levels in MDA-MB-231 and MDA-MB-468 cells and the normal human breast epithelial MCF-10A cells, 2′,7′-dichlorodihydrofluorescein diacetate (DCFDA) was used in the assay. Cells were seeded at 20,000 cells/well in black 96-well flat clear bottom tissue-culture plates. After overnight culture at 37 ºC, cells were incubated with DCFDA (25 µM) in Hanks Balanced Salt Solution (HBSS, ThermoFisher Scientific, Waltham, MA) at 37 ºC for 45 min. The cells in culture were subsequently washed with PBS and untreated (DMSO, control) or treated with R001 or other compounds at different concentrations for different times. Fluorescence signal was measured at 485/535 nm.

CellROX Deep Red Flow Cytometry Assay Kit (Thermo Fisher Scientific, #C10491) was used to further measure cellular ROS levels in cells. Briefly, cells were seeded in 6-well culture plates. After overnight incubation, the cells were treated with or without R001 for 16 h. Tert-butyl hydroperoxide (TBHP) were used as a positive control to treat cells at 200 µM for 30 min. Cells were subsequently harvested and stained with CellROX Deep Red reagent by incubating with the dye at a final concentration of 500 nM for 30 min at 37 °C in the dark. The samples were then immediately analyzed by FACS flow cytometry (BD Biosciences) using an APC filter. Approximately 2 × 10^4^ cells were analyzed in each of the samples. Mean fluorescence intensity (MFI) value analyzed was performed using FlowJo V10 software.

Hydrogen peroxide (H_2_O_2_) release was assayed using the Amplex Red Hydrogen Peroxide/Peroxidase Assay Kit (Thermo Fisher Scientific) according to the instructions with minor modifications. Briefly, cells were seeded in 96-well plates and allowed to culture overnight. Next day, the culture media was removed and the cells were washed once with Krebs-Ringer phosphate (KRPG) buffer consisting of 145 mM NaCl, 5.7 mM sodium phosphate, 4.86 mM KCl, 0.54 mM CaCl2, 1,22 mM MgSO4, 5.5 mM glucose, pH 7.35). Appropriate dilutions of DMSO, R001 or H_2_O_2_ standard in KRPG buffer were added to the cells at 50 μL per well for 0-48 h. Subsequently, the Amplex Red reaction mixture containing 100 μM Amplex Red reagent and 0.2 U/mL horseradish peroxidase in KRPG buffer was added at 50 μL to each well, and the fluorescent intensity was determined using SpectraMax ID5 microplate reader with excitation at 535 nm and emission at 590 nm at the indicated time points. Results were normalized to the fold change of control samples.

Mitochondrial superoxide production was monitored by MitoSOX Deep Red staining (#MT14-12, Dojindo, Japan) following the manufacturer’s protocol. Briefly, cells were seeded in 6-well plates (2 x 10^5^ cells per well) and cultured overnight, and then treated with R001 at the indicated time and concentration. Subsequently, the supernatant was removed and the cells were washed two times with Hanks' Balanced Salt Solution (HBSS), and the working solutions of MitoSOX red (10 μM) and Hoechst33342 (1 μg/ml) were added, and the cells were further incubated for 30 min at 37 °C in the dark. Thereafter, the cells were washed with HBSS and observed under a fluorescence microscope (KEYENCE, IL).

*Seahorse assay*- Mitochondrial respiration was measured with Agilent Seahorse XFe96/XF Pro according to the manufacturer’s instructions. Cells were plated in a Seahorse XF96 cell culture plate at a density of 2×10^4^ cells/well for overnight culture and then treated with R001 (5 µM) for 3 h at normal condition. The culture media was then exchanged with Agilent Seahorse XF Media (DMEM supplemented with 1 mM pyruvate, 2 mM glutamine and 10 mM glucose), and the culture was equilibrated in a 37°C incubator without CO_2_ for 1 h prior to the assay. Cellular oxygen consumption was monitored at basal condition and after injection of optimal concentrations of oligomycin, FCCP and Rotenone plus Antimycin A. At least 4 replicate wells were used for each group.

*Comet assay for DNA damage*- Cells in culture were untreated (DMSO, control) or treated with R001 at the indicated concentrations for 24 h, or in the case of H_2_O_2_ (400 µM), cells were treated for 1 h. DNA damage was measured by the OxiSelect Comet Assay Kit (Cell Biolabs Inc, San Diego, CA) according to manufacturer’s instructions with all operations conducted under dimmed light to prevent the occurrence of additional DNA damage. Briefly, following treatment, cells were trypsinized, collected, and resuspended at 1 x 10^5^ cells/ml in ice-cold PBS. The resuspended cells were mixed with Comet Agarose at 1:10 ratio (v/v), and the mixture was immediately transferred onto the top of the base layer prepared on the slides. The slides were then immersed in the provided lysis buffer at 4 ºC in the dark for 60 min and subjected to electrophoresis in an alkaline buffer for 30 min at the voltage of 1 V/cm according to the instructions. The slides were washed with water, fixed in 70% ethanol and incubated with diluted Vista Green DNA Dye for 15 min at room temperature. Images were taken under an Olympus inverted microscope with epifluorescence capability using a FITC filter. The percentage of DNA in the tail for each cell was calculated by CASPlab software ^10^. At least 25 cells were analyzed for each treatment.

*3D spheroid assay-* MDA-MB-231 cells (2,000 cells/well) were seeded in ultra-low attachment 96-well round bottom plates, incubated for 48 h, and treated with DMSO, 2.5 or 5 µM R001. After 24 h of treatment, matrigel was added. The spheroids were visualized under an Axiovert 200 inverted fluorescence microscope (Zeiss, Germany) every 24 h. Pictures were taken using a mounted Canon Powershot A640 digital camera (Melville, NY) at every 24 h for up to 72 h at a 5x or 10x magnification.

*Scratch assay for migration-* This assay was performed as previously reported ^8^. Briefly, sub-confluent cultures of MDA-MB-231 and MDA-MB-468 cells in 6-well plates were wounded using pipette tips, treated with compound or vehicle (DMSO), and allowed to migrate into the denuded area over 22 and 40 h periods. The migration of cells was visualized at a 10X magnification using an Axiovert 200 inverted fluorescence microscope (Zeiss, Germany), with pictures taken using a mounted Canon Powershot A640 digital camera. The wound width was measured by ImageJ, and the relative wound width was calculated compared to the time 0.

*Mice and in vivo tumor studies-* Mice were housed in specific pathogen-free conditions in the animal facility. All animal experiments were conducted in accordance with the recommendations in the Guide for the Care and Use of Laboratory Animals on a protocol approved by the Institutional Animal Care and Use Committee (IACUC). Five-week-old female athymic nude mice were purchased from Envigo (Indianapolis, IN) and maintained in the institutional animal facilities approved by the American Association for Accreditation of Laboratory Animal Care. Subcutaneous xenograft studies were performed as previously reported ^6, 8^. Mice were injected subcutaneously in the right flank area with 7.3 x 10^6^ MDA-MB-468 cells in 100 μL PBS. When an average tumor volume of 100 mm^3^ was established, tumor-bearing mice were grouped so that the mean tumor sizes in all groups were nearly identical. Mice were administered R001 (5 mg/kg, oral gavage, 100 μL PBS with 5% DMSO) every day, 5 times per week for 75 days. For the R001 combination with NAC treatment, NAC was orally given alone (120 mg/kg) ^11^, or combined with R001 (5 mg/kg) every day, 5 times per week for 60 days. Animals were monitored daily, tumor size was measured with calipers and body weight was taken every 3-4 days. Tumor volumes were calculated according to the formula *V = 0.52a^2^b*, where a is the smallest superficial diameter and b is the largest superficial diameter. For each treatment group, the tumor volumes for each set of measurements were statistically analyzed in comparison with the control (vehicle-treated) group using *Student*’s *t* test.

*Immunohistochemistry (IHC)*- IHC was performed through Cedars Sinai Biobank and Research Pathology Resource core facility on a Discovery Ultra staining system (Roche Tissue Diagnostics, Ventana Medical Systems, Inc.). The samples used for immunohistochemical analysis came from mice tumor tissues which were treated with or without R001 (5 mg/kg) for 75 days. Formalin-fixed, paraffin-embedded tissue blocks were cut into serial 4 µm sections. The sections were deparaffinized at 72 ^o^C with EZ solution (Roche Diagnostics). After antigen retrieval and endogenic peroxidase block, the primary antibody (Rabbit anti-8-OHdG, 1:500 (Bioss Inc)) was applied and incubated for 1 h at room temperature. Subsequently, DISCOVERY anti-Rabbit HQ (Roche, #760-4815) and anti-HQ HRP (Roche, #760-4820) detection was performed, and then DISCOVERY ChromoMap DAB Kit (Roche, #760-159,) were applied. After Mayer’s hematoxylin nuclear counterstain, slides were mounted with coverslip with mounting medium. The images were acquired using a fluorescence microscope (KEYENCE, IL). Three mice from each group were included in the analysis.

References

1. Youn UJ, Miklossy G, Chai X, Wongwiwatthananukit S, Toyama O, Songsak T, et al. Bioactive sesquiterpene lactones and other compounds isolated from Vernonia cinerea. Fitoterapia. 2014;93:194-200.

2. Miklossy G, Youn UJ, Yue P, Zhang M, Chen C-H, Hilliard TS, et al. Hirsutinolide series inhibit Stat3 activity, modulate GCN1, MAP1B, Hsp105, G6PD, Vimentin, and importin a-2 expression, and induce antitumor effects against human glioma. J Med Chem. 2015;58:7734-7748.

3. Jakupovic J, Banerjee S, Castro V, Bohlmann F, Schuster A, Msonthi JD, et al. Poskeanolide, a seco-germacranolide and other sesquiterpene lactones from Vernonia species. Phytochemistry. 1986;25:1359–1364.

4. Garcia R, Bowman TL, Niu G, Yu H, Minton S, Muro-Cacho CA, et al. Constitutive activation of Stat3 by the Src and JAK tyrosine kinases participates in growth regulation of human breast carcinoma cells. Oncogene. 2001;20:2499-2513.

5. Turkson J, Ryan D, Kim JS, Zhang Y, Chen Z, Haura E, et al. Phosphotyrosyl peptides block Stat3-mediated DNA binding activity, gene regulation, and cell transformation. J Biol Chem. 2001;276:45443-45455.

6. Yue P, Lopez-Tapia F, Paladino D, Li Y, Chen C-H, Hilliard T, et al. Hydroxamic acid and benzoic acid-based Stat3 inhibitors suppress human glioma and breast cancer phenotypes in vitro and in vivo. Cancer Res. 2016;76:652-663.

7. Yue P, Zhu Y, Brotherton-Pleiss C, Fu W, Verma N, Chen J, et al. Novel potent azetidine-based compounds irreversibly inhibit Stat3 activation and induce antitumor response against human breast tumor growth in vivo. Cancer Lett. 2022;534:215613.

8. Zhang X, Yue P, Page BD, Li T, Zhao W, Namanja AT, et al. Orally bioavailable small-molecule inhibitor of transcription factor Stat3 regresses human breast and lung cancer xenografts. Proc Natl Acad Sci U S A. 2012;109:9623-9628.

9. Zhang X, Yue P, Fletcher S, Zhao W, Gunning PT, Turkson J. A novel small-molecule disrupts Stat3 SH2 domain-phosphotyrosine interactions and Stat3-dependent tumor processes. Biochem Pharmacol. 2010;79:1398-1409.

10. Lu Y, Liu Y, Yang C. Evaluating In Vitro DNA Damage Using Comet Assay. J Vis Exp. 2017;(128).

11. Zhang VX, Sze KM, Chan LK, Ho DW, Tsui YM, Chiu YT, et al. Antioxidant supplements promote tumor formation and growth and confer drug resistance in hepatocellular carcinoma by reducing intracellular ROS and induction of TMBIM1. Cell Biosci. 2021;11:217.

**Supplementary Figures**

**Figure S1. Effects of R001 in colony survival and wound-healing assays.** (A) Single-cell cultures of MDA-MB-468, MDA-MB-231, and MCF-10A cells treated once with 0-1.0 μM R001 and allowed to grow until large colonies were visible, which were stained with crystal violet and imaged; and (B) Images of cultured MDA-MB-468 and MDA-MB-231 cells, which were wounded, treated once with 0-5 µM R001 and allowed to migrate to the denuded area over 22-40 h. Control lane (0, -) represents cells treated with 0.1% DMSO. Data are representative of 2-3 independent determinations.

**Figure S2. Effects of R001 on** **6-Phosphogluconate dehydrogenase (6PGD), malic enzymes (ME) 2 and 3, and** **methylenetetrahydrofolate dehydrogenases (MTHFD) 1 and 2 expression**. Immunoblotting analysis of whole-cell lysates of equal total protein prepared from the MDA-MB-231 or MDA-MB-468 cells untreated or treated with R001 5 µM for 3 or 16 h and probing for 6PGD, ME2, ME3, MTHFD1, MTHFD2 or tubulin. Positions of proteins in gel are labeled; control lane (C) represents whole-cell lysates prepared from 0.1% DMSO-treated cells. Data are representative of 2 independent determinations. MW, molecular weight.

**Figure S3. R001 treatment of tumor cells induces ROS production.** MDA-MB-231, MDA-MB-468, or MCF-10A lines were untreated or treated with 5 µM R001 alone for 16 h or with 100 µM tert-Butyl hydroperoxide  (TBHP) for 30 min. Cells were processed and stained for reactive oxygen species (ROS) levels with CellRox Deep Red and quantified (A) or MitoSox and Hoechst 33342 dyes and imaged (B). Values, mean ± SD, n=3. **p* <0.05 and ***p* <0.01. Data are representative of 2 independent determinations.

**Figure S4. Effects of R001 treatment on the induction of hydrogen peroxide.** Breast cancer, MDA-MB-231 and MDA-MB-468 cells or normal human breast epithelial, MCF-10A line in culture were untreated (Con) or treated once with (A) 2.5-10 µM R001 for the indicated times and cells were prepared and assayed for (A) H_2_O_2_ levels, or (B) 20 µM hydrogen peroxide (H_2_O_2_) for 24 h and samples were prepared and assayed for H_2_O_2_ levels. Control lane (Control) represents 0.1% DMSO-treated cells. Values, mean ± S.D., n=5. **p* <0.05, ***p* <0.01, and ****p* <0.001.

**Figure S5. Fluorescent microscopy images of propidium iodide (PI) staining of MDA-MB-231 cells treated with R001.** Cells were treated with 10 µM R001 for 24 h, stained with PI and imaged under phase-contrast microscopy. Data are representative of 2 independent determinations.

**Figure S6. Measurement of PARP and caspase 3 cleavage induced by R001.** MDA-MB-468 and MDA-MB-231 cells in culture were untreated or treated with R001, 0.5-5 µM for 3 h, or 2.5 or 5 µM for 0.5-48 h and whole-cell lysates were prepared and subjected to immunoblotting probing for poly (ADP-ribose) polymerase (PARP), cleaved PARP, caspase-3, cleaved caspase-3 and tubulin. Positions of proteins in gel are labeled; control lane (0) represents whole-cell lysates prepared from 0.1% DMSO-treated cells. Data are representative of 2 independent determinations. MW, molecular weight.

**Figure S7. Effects of R001 on GSH and NADPH levels and TrxR1 and G6PD activities in tissues from human triple-negative breast tumor xenografts *in vivo*.** Mice bearing MDA-MB-468 subcutaneous tumor xenografts were administered 5 mg/kg R001 via oral gavage, or vehicle (5% DMSO in PBS) every day, 5 days per week for 75 days. Tumors were excised at the end of study and the tumor tissues were analyzed for GSH and NADPH levels, or TrxR1 and G6PD activities, which are plotted for each individual tumor tissue. n=6 or 7.

**Figure S8. STAT3, G6PD, and TrxR1 gene expression in human breast cancer compared to normal tissue and the correlation between the expression levels and overall survival in patients not previously treated. (A)** Gene expression of STAT3, G6PD, and TrxR in the TCGA and GEO databases for normal and breast tumor tissues samples. **(B)** Untreated breast cancer patients were grouped into high (red) and low (black) groups of STAT3, G6PD, or TrxR expression and Kaplan-Meier survival curves were plotted for each group of patients.
