# Supplementary material for: Natural product preferentially targets redox and metabolic adaptations and aberrantly active STAT3 to inhibit breast tumor growth in vivo
Source: Cell Death Dis. 2022 Dec 6;13(12):1022. doi: 10.1038/s41419-022-05477-2 (PMC9726930; doi:10.1038/s41419-022-05477-2)

Supplementary Figures

Fig. S1.

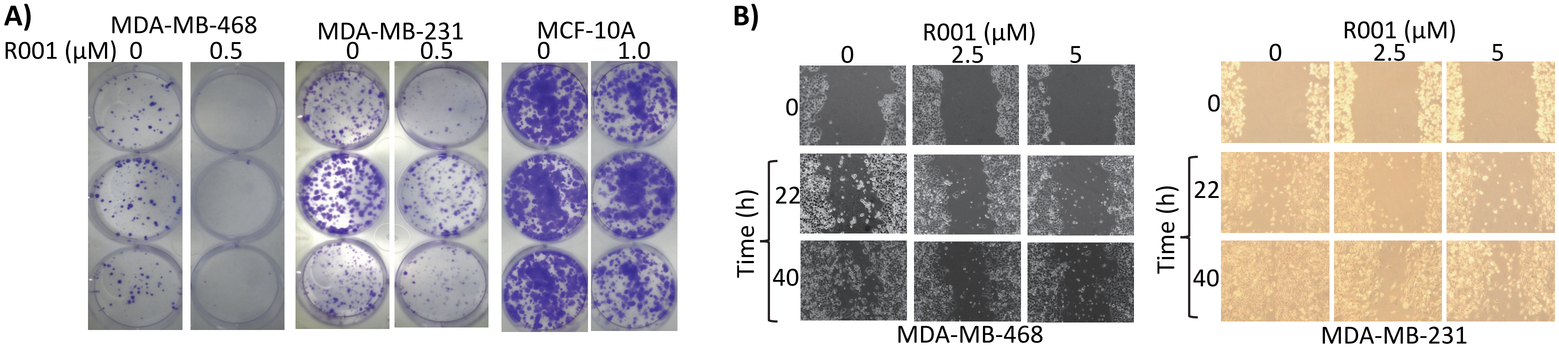

Supplementary Figures

Fig. S2.

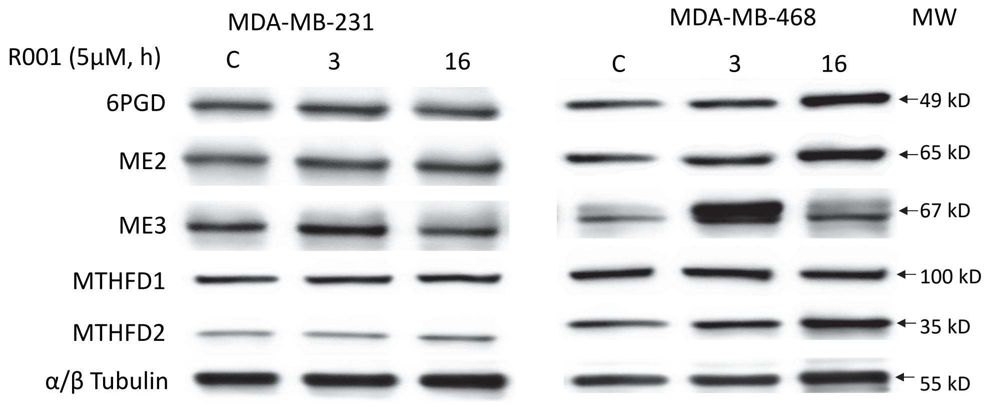

Fig. S3. Supplementary Figures

A) Quantified ROS production by staining with CellRox DeepRed

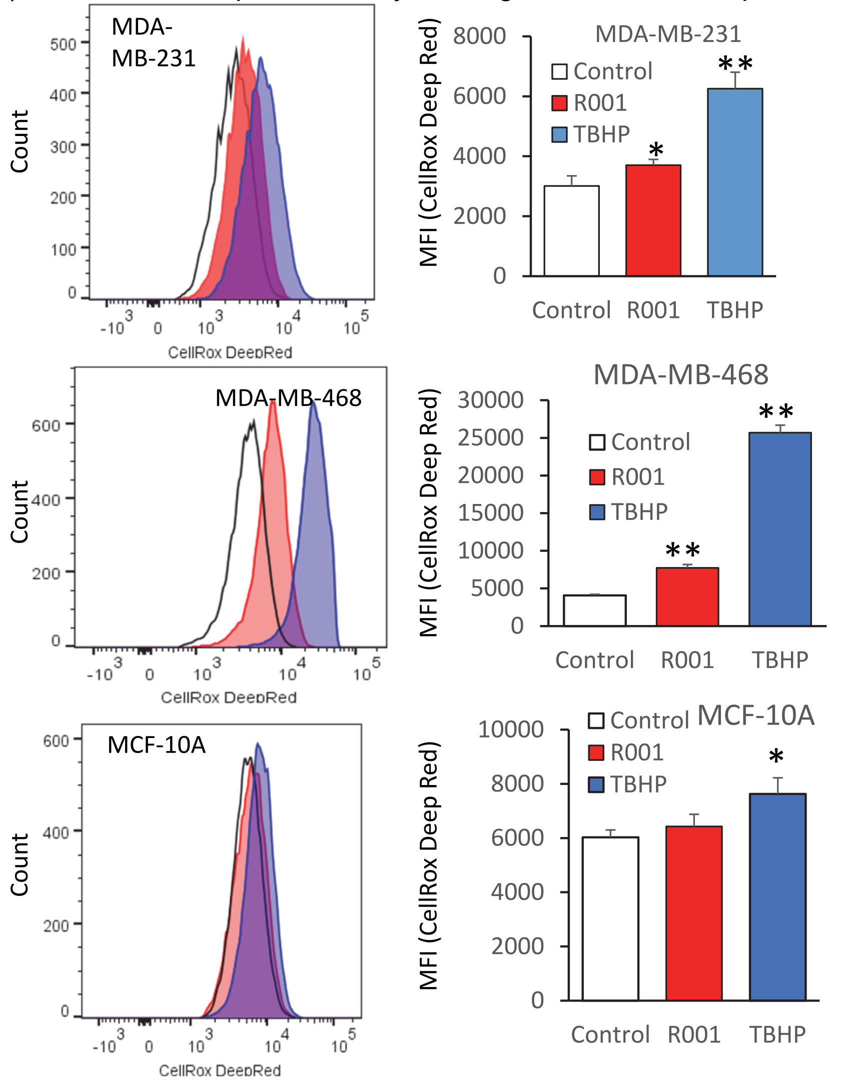

B) Quantification of the source of ROS

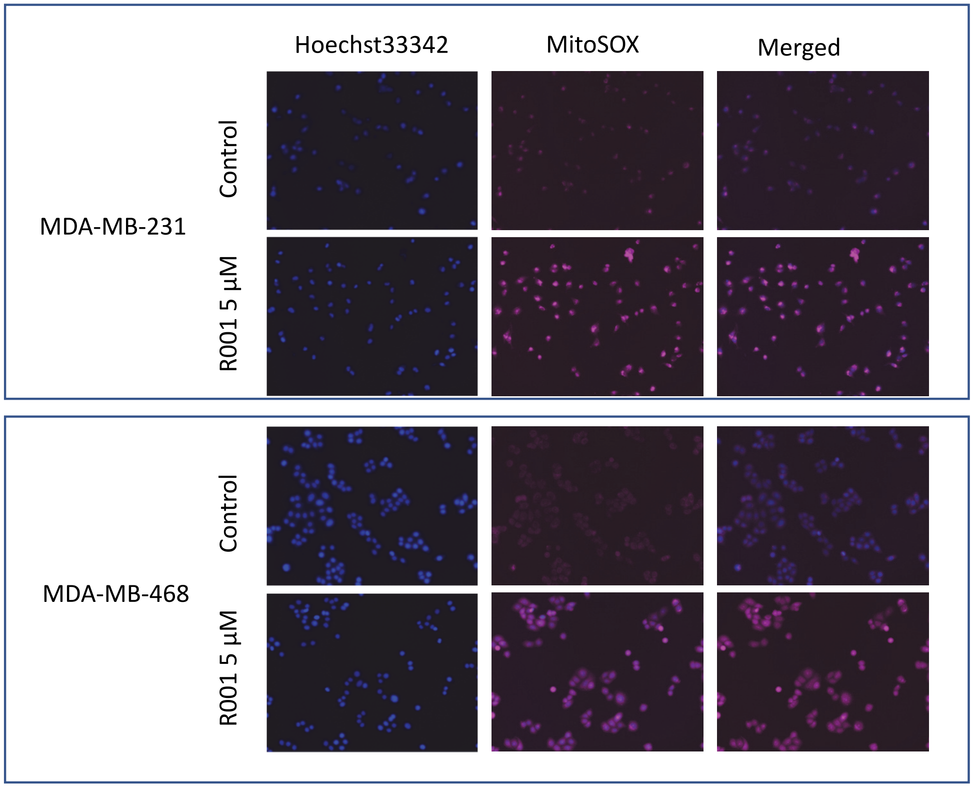

Supplementary Figures

Fig. S4.

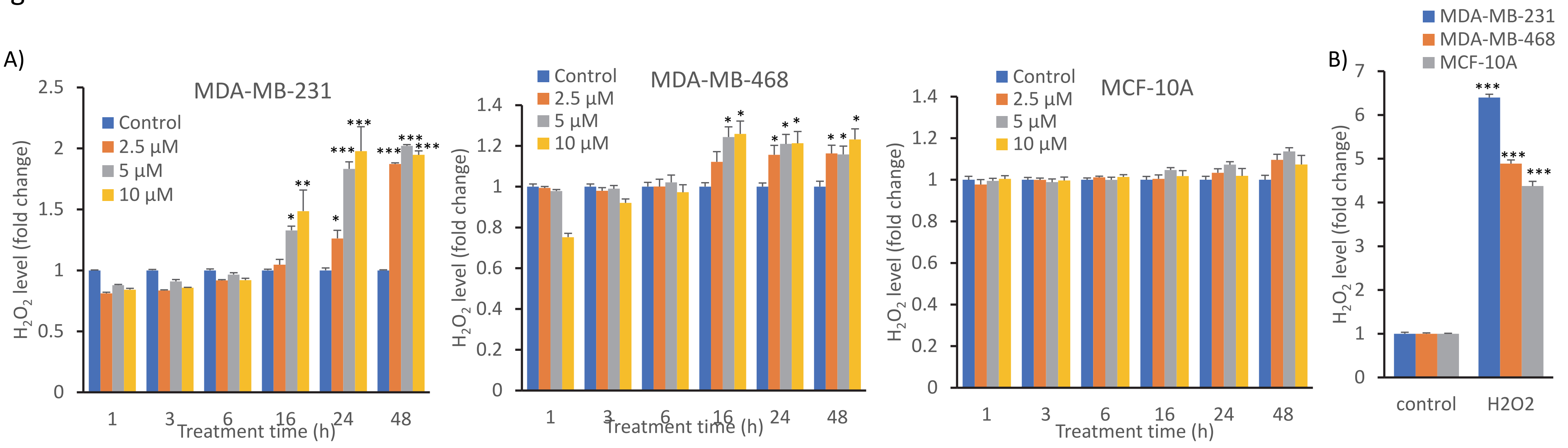

Supplementary Figures

Fig. S5.

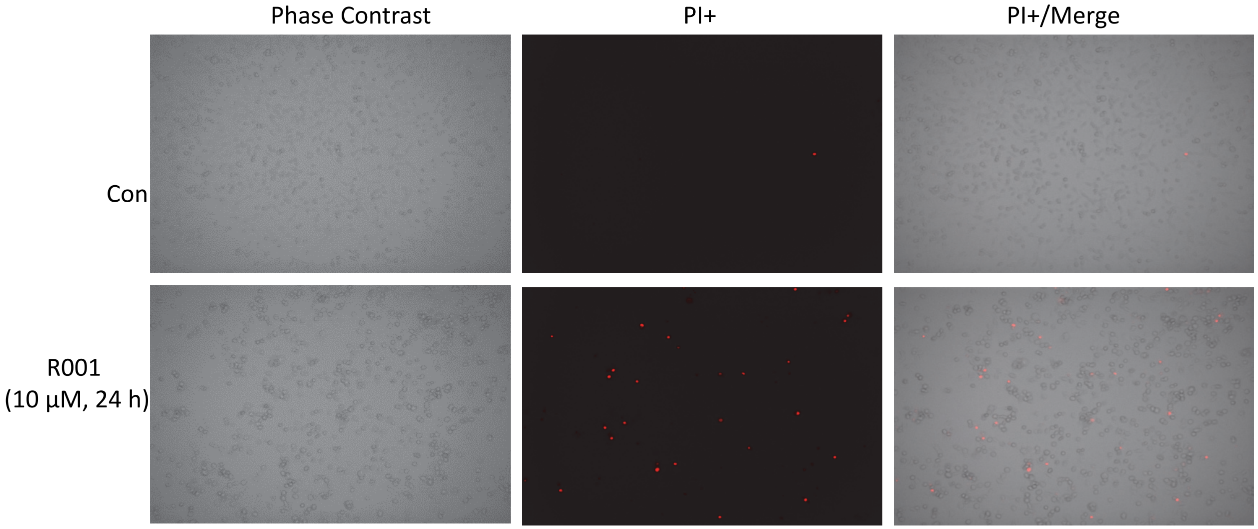

Supplementary Figures

Fig. S6.

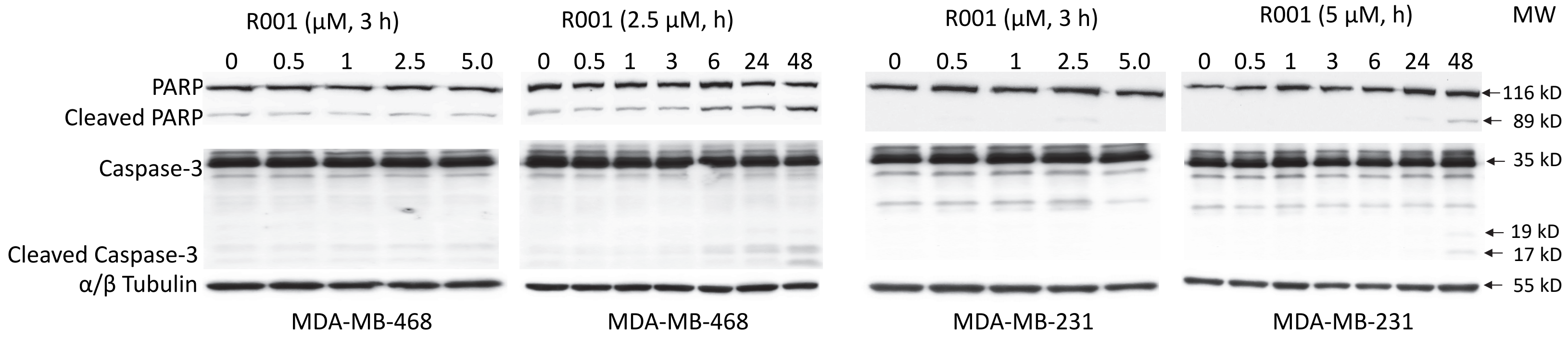

Supplementary Figures

Fig. S7.

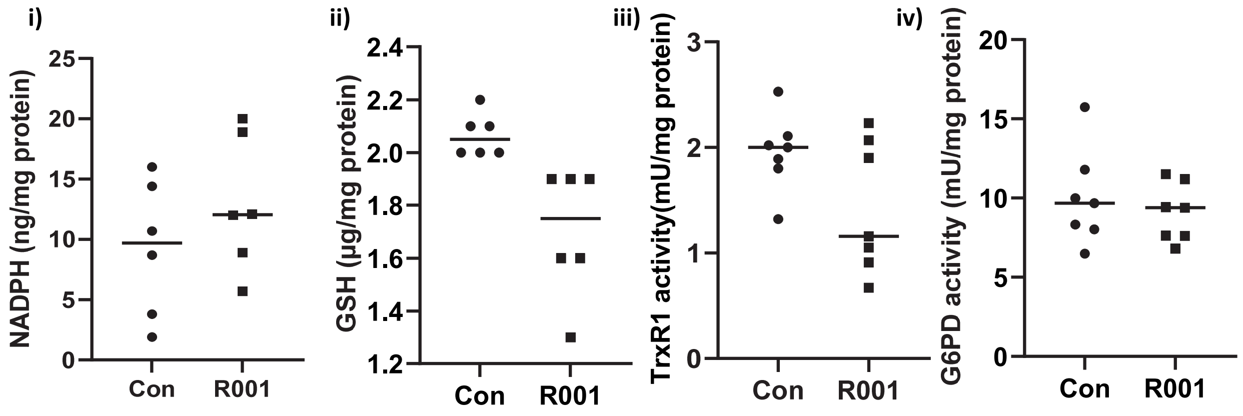

Supplementary Figures

Fig. S8.

A)

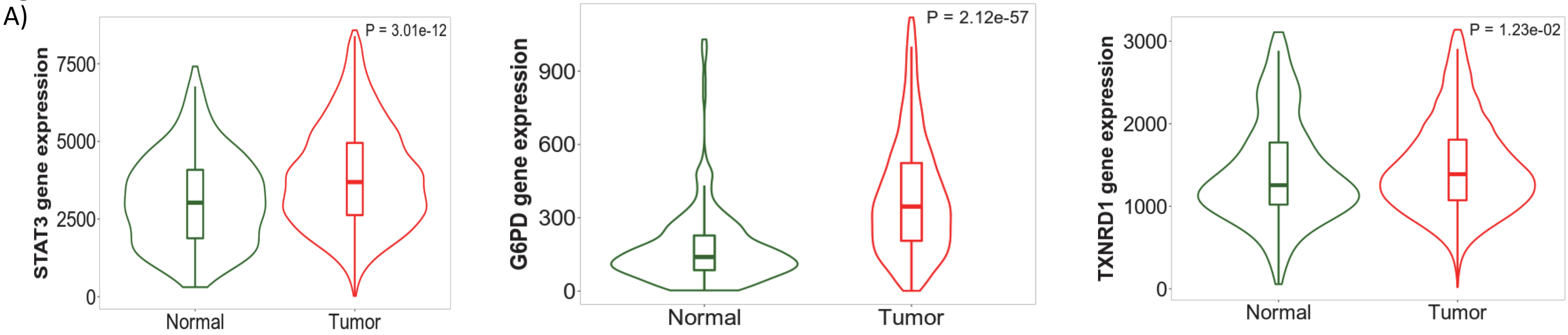

B)

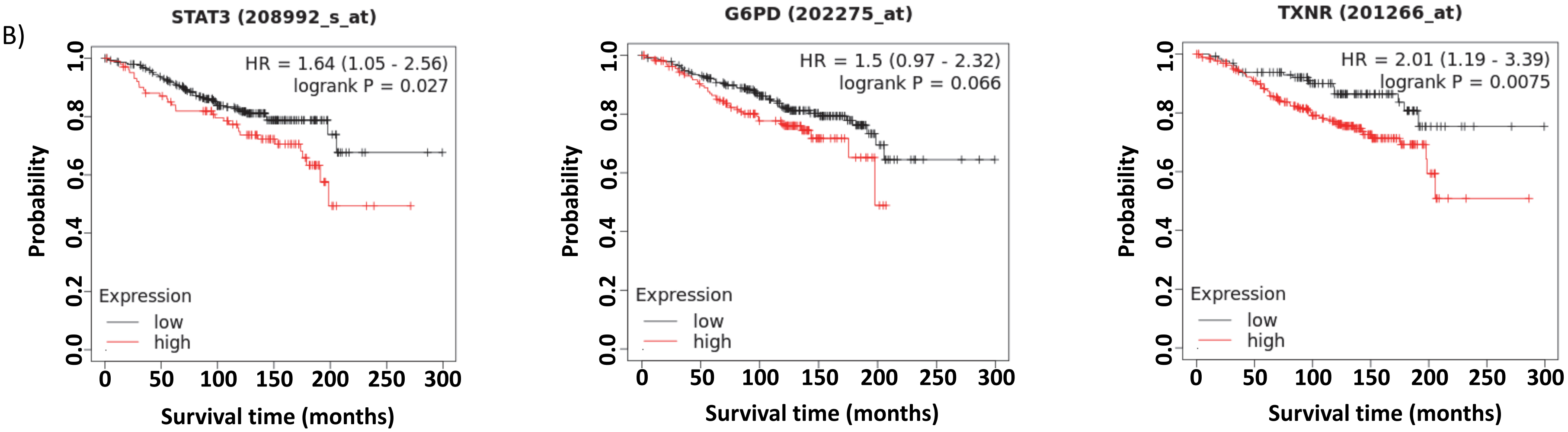

Supplement: Supplementary file 2 — Supplementary Materials [file 41419_2022_5477_MOESM2_ESM.pdf]
