# Supplementary material for: Natural product preferentially targets redox and metabolic adaptations and aberrantly active STAT3 to inhibit breast tumor growth in vivo
Source: Cell Death Dis. 2022 Dec 6;13(12):1022. doi: 10.1038/s41419-022-05477-2 (PMC9726930; doi:10.1038/s41419-022-05477-2)
Supplement: Supplementary file 5 — Reagents List [file 41419_2022_5477_MOESM5_ESM.pdf]

Antibody

1. P-Stat3 (Y705) (3E2) mouse mAb, Cell Signaling Technology, Inc., Cat # 9138L, Lot 9;
2. P-Stat3 (Y705) (D3A7) XP Rabbit mAb, Cell Signaling Technology, Inc., Cat # 9145L, Lot 34;
3. P-Stat3 (S727) antibody, Cell Signaling Technology, Inc., Cat # 9134S, Lot 21;
4. Stat3 (124H6) mouse mAb, Cell Signaling Technology, Inc., Cat # 9139S, Lot 10, 12;
5. Stat3 (79D7) Rabbit mAb, Cell Signaling Technology, Inc., Cat # 4904S, Lot 7;
6. GAPDH (C65), Cat # sc-32233, Lot # H1213, mouse monoclonal IgG1, Santa Cruz Biotech;
7. P-EGF Receptor (Y1068) Rabbit Ab, Cell Signaling Technology, Inc., Cat # 2220S, Lot 2;
8. EGF Receptor Rabbit Ab, Cell Signaling Technology, Inc., Cat # 2232S, Lot 15;
9. P-Jak2 (Y1007/1008) (C80C3) Rabbit mAb, Cell Signaling Technology, Inc., Cat # 3776S, Lot 11;
10. Jak2 (D2E12) XP(R) Rabbit mAb, Cell Signaling Technology, Inc., Cat # 3230S, lot 8;
11. G6PD Antibody (H-160), rabbit polyclonal IgG, Santa Cruz Biotech, Cat # sc-67165, Lot # F1011;
12. Anti-TrxR1 (B-2) antibody, mouse monoclonal IgG2a, Santa Cruz Biotech, Cat # sc-28321, Lot # C1813;
13. c-Myc Rabbit Ab, Cell Signaling Technology, Inc., Cat # 9402S, Lot 7;
14. VEGF (147) rabbit polyclonal IgG, Santa Cruz Biotech, sc-507, Lot # L0811;
15. PARP Rabbit Ab, Cell Signaling Technology, Inc., Cat # 9542, Lot 6;
16. Caspase 3 (8G10) Rabbit mAb, Cell Signaling Technology, Inc., Cat # 9665S, Lot 6;
17. α/β-Tubulin Antibody, Cell Signaling Technology, Inc, Cat #2148S, Lot 8,;
18. p21 Waf1/Cip1 (12D1) Rabbit mAb, Cat #2947S, Lot 1, Cell Signaling Technology, Inc;
19. RIF1 (D2F2M) Rabbit mAb, Cell Signaling Technology, Inc.,Cat #95558S, Lot 1;
20. Phospho-ATM (Ser1981) (D6H9) Rabbit mAb, Cell Signaling Technology, Inc., Cat #5883T, Lot 6;
21. ATM (D2E2) Rabbit mAb, Cell Signaling Technology, Inc., #2873T, Lot 5;
22. Gasdermin D (E8G3F) Rabbit mAb, Cell Signaling Technology, Inc., Cat #97558T, Lot 1;

23. Cleaved Gasdermin D (Asp275) (E7H9G) Rabbit mAb, Cell Signaling Technology, Inc, Cat # 36425T, Lot 3;
24. Caspase-1 (D7F10) Rabbit mAb, Cell Signaling Technology, Inc., Cat #3866T, Lot 4;
25. Cleaved Caspase-1 (Asp297) (D57A2) Rabbit mAb, Cell Signaling Technology, Inc., Cat #4199T, Lot 4;
26. IL-1β (D3U3E) Rabbit mAb, Cell Signaling Technology, Inc., Cat #12703T, Lot 5;
27. Cleaved-IL-1β (Asp116) (D3A3Z) Rabbit mAb, Cell Signaling Technology, Inc., Cat #83186T, Lot 3;
28. Phospho-Cyclin B1 (Ser133) (9E3) Rabbit mAb, Cell Signaling Technology, Inc., Cat #4133S, Lot3 ;
29. Cyclin B1 rabbit monoclonal antibody, Novus Biologicals, Cat # NBP1-61243, Lot # 210068;
30. Phospho-cdc2 (CDK1, Tyr15) (10A11) Rabbit mAb, Cell Signaling Technology, Inc., Cat #4539T, Lot 2;
31. CDK1 antibody: cdc2 (POH1) Mouse mAb, Cell Signaling Technology, Inc., Cat #9116S, Lot 7;
32. Recombinant Anti-Cyclin D1 antibody [SP4], Abcam, Cat # ab16663, Lot # GR3256069-11;
33. PLK1 (208G4) Rabbit mAb, Cell Signaling Technology, Inc., Cat #4513S, Lot 4;
34. Chk2 (D9C6) XP Rabbit mAb, Cell Signaling Technology, Inc., Cat #6334T, Lot 5;
35. Histone H2A.X (D17A3) XP® Rabbit mAb, Cell Signaling Technology, Inc., Cat #7631T, Lot 6;
36. cdc25C (5H9) Rabbit mAb, Cell Signaling Technology, Inc., Cat #4688T, Lot 3;
37. Mcl-1 rabbit mAb, Cell Signaling Technology, Inc., Cat # 4572, Lot 3;
38. Bcl-2 (D55G8) Rabbit mAb, Cell Signaling Technology, Inc., Cat # 4223s, Lot 4;
39. Anti-Mouse IgG (H+L), HRP Conjugate, Promega, Cat # W4021;
40. Anti-Rabbit IgG (H+L), HRP Conjugate, Promega, Cat # W4011;
41. 6PGD rabbit Ab, Cell Signaling Technology, Inc., Cat # 13389S, Lot 1;
42. MTHFD2 rabbit mAb, Cell Signaling Technology, Inc., Cat # 41377S, Lot 1;
43. ME2 XP® rabbit mAb, Cell Signaling Technology, Inc., Cat # 15506T, Lot 1;
44. ME3 rabbit mAb, Cell Signaling Technology, Inc., Cat # 18457S, Lot 1;

45. MTHFD1 (A-8) mouse monoclonal IgG, Santa Cruz Biotech, Cat # sc-271412, Lot # B0421;
46. 8-OHdG polyclonal antibody, Bioss Inc, Cat # bs-1278R, Lot # BB04226515

Reagents and assay kits

1. N-Acetyl-L-cysteine (NAC), Sigma-Aldrich, Cat # A9164, Lot# SLCB5087;
2. L-Glutathione Reduced (GSH), Sigma-Aldrich, Cat # G4251, Lot# SLCF2362;
3. β-Nicotinamide adenine dinucleotide 2′-phosphate reduced tetrasodium salt hydrate (NADPH), Sigma-Aldrich, Cat # N75005, Lot# SLCH1125;
4. Insulin from bovine pancreas, Sigma-Aldrich, Cat # I6634, Lot# SLBN1822;
5. Deferoxamine mesylate salt, Sigma-Aldrich, Cat # D9533, Lot# BCCB3792;
6. 2′,7′-Dichlorofluorescin diacetate (DCFDA), Sigma-Aldrich, Cat # D6883, Lot# 059M4133V;
7. L- Buthionine Sulfoximine (BSO), EMD Millipore, Corp., Cat # 5.08228.0001, Lot# 3559029;
8. Recombinant Human Thioredoxin / Trx protein, Abcam, Cat # ab283443, Lot # GR3399000-1;
9. Matrigel® Basement Membrane Matrix, Corning, Cat # 356234, Lot # 308002;
10. NADP/NADPH Quantitation Kit, Sigma-Aldrich, Cat # MAK038, Lot# 7E20K03470;
11. Glucose-6-Phosphate Dehydrogenase Activity Assay Kit, Sigma-Aldrich, Cat # MAK015, Lot# 6D22K07570;
12. Reduced glutathione assay kit, Sigma-Aldrich, Cat # MAK364, Lot# 7B05K04640;
13. Lipopolysaccharides (LPS), Cell Signaling Technology, Inc., Cat # 14011S, Lot ;
14. Recombinant Human Thioredoxin / Trx protein, Abcam, Cat # ab283443, Lot # GR3399000-1;
15. OxiselectTM-comet assay kit, Cell Biolabs, INC., Cat # STA-350, Lot # 60220211;
16. GSH/GSSG assay kit, Sigma-Aldrich, Cat # MAK440, Lot# 440CC04A26;
17. Mitochondrial Superoxide Detection kit, Dojindo, Cat # MT14, Lot# VA750;
18. CellROX Deep Red Flow Cytometry Assay Kit, Thermo Fisher Scientific, Cat # C10491, Lot# 2497563
19. Agilent Seahorse XFe96/XF Pro Extracellular Flux Assay kit, Agilent Technologies, Cat # 102416, Lot# W36321
